# Unraveling the role of the WIPF1/ACTN4 complex in podosome formation of human placental EVTs: Insights into recurrent spontaneous abortion

**DOI:** 10.1016/j.gendis.2025.101665

**Published:** 2025-05-02

**Authors:** Cong Li, Shengya Wang, Jing Tang, Xin Luo, Luxing Ge, Youlong Xie, Lijuan Fu, Lingling Ruan, Enoch Appiah Adu-Gyamfi, Fangfang Li, Yingxiong Wang, Hongbo Qi, Yubin Ding

**Affiliations:** a Department of Obstetrics and Gynecology, Women and Children's Hospital of Chongqing Medical University, Chongqing 401147, China; b Joint International Research Laboratory of Reproduction and Development of the Ministry of Education of China, School of Public Health, Chongqing Medical University, Chongqing 401147, China; c Department of Bioinformatics, School of Basic Medicine, Chongqing Medical University, Chongqing 400016, China; d Department of Obstetrics and Gynecology, The First Affiliated Hospital of Chongqing Medical University, Chongqing 400016, China; e Department of Biomedical Sciences, State University of New York at Albany, Albany, NY 12222, USA; f Department of Pharmacology, Academician Workstation, School of Pharmacology, Changsha Medical University, Changsha, Hunan 410219, China

**Keywords:** Extravillous trophoblasts, Invasion, Podosome formation, Recurrentspontaneous abortion, WIPF1

## Abstract

Successful placental development and pregnancy rely on effective extravillous trophoblast (EVT) invasion. The mechanisms underlying inadequate EVT invasion in recurrent spontaneous abortion (RSA) remain unclear. WAS/WASL interacting protein family member 1 (WIPF1), the key regulator of cytoskeletal dynamics, is exclusively expressed in first-trimester placental EVTs. Knockdown experiments revealed WIPF1's crucial involvement in successful placental development; reduced levels impaired cell migration, while overexpression induced the opposite effects. Moreover, WIPF1 knockdown in hTSC-derived EVTs hampered trophoblast differentiation. WIPF1 interacted with ACTN4 to regulate podosome formation, matrix degradation, and actin polymerization, potentially mediated by its ARG54 site. Notably, WIPF1 was significantly down-regulated in human RSA patient EVTs and RSA mice trophoblast giant cells (CBA/J × DBA/2). This association suggests WIPF1 as a potential key player in RSA pathogenesis. In conclusion, our study spotlights WIPF1 as a pivotal factor in EVT invasion, emphasizing its multifaceted roles and implications in pregnancy complications like RSA.

## Introduction

The human placenta is a unique, transient organ that plays a critical role in supporting pregnancy and fetal development. It is responsible for a variety of essential functions, including supplying oxygen and nutrients to the fetus, removing metabolic waste, producing vital hormones, and modulating the maternal immune response to ensure the protection of the semi-allogeneic fetus. These functions are crucial for the successful progression of pregnancy and the health of both mother and baby.^1^ During early placental development, the trophectoderm cells, which form the outer layer of the blastocyst, proliferate and differentiate into cytotrophoblast cells (CTBs). These CTBs act as progenitor cells with the ability to self-renew, giving rise to two major trophoblast lineages: villous trophoblasts and extravillous trophoblasts (EVTs). Villous trophoblasts form the outer layer of the placental villi, facilitating gas and nutrient exchange between the mother and fetus. EVTs, on the other hand, are specialized cells that migrate from the chorionic villi into the maternal decidua and spiral arteries, contributing to the remodeling of maternal vessels to ensure adequate blood supply to the placenta.^2^

The invasion and remodeling by EVTs are crucial for placental development and pregnancy health. This process is tightly regulated by molecular pathways, including matrix metalloproteinases, which degrade the extracellular matrix to promote EVT invasion, and integrins, which guide adhesion and migration.^3^ Growth factors and cytokines, like transforming growth factor-beta (TGF-β) and vascular endothelial growth factor (VEGF),^4^ balance invasion regulation, while the WNT and NOTCH signaling pathways control migration and survival.^5^ EVTs also express human leukocyte antigen-G (HLA-G) to avoid immune detection, ensuring tolerance of the semi-allogeneic fetus.^6^ Any disruption in their migration or function may result in serious pregnancy complications, such as preeclampsia, fetal growth restriction, or even miscarriage.^7^, ^8^, ^9^, ^10^

Single-cell RNA sequencing has emerged as a powerful tool for identifying cell-type-specific transcriptomic signatures, offering critical insights into gene involvement in trophoblast proliferation and differentiation, particularly in EVTs during both physiological and pathological pregnancies.^11^^,^^12^ Numerous genes, including achaete-scute family bHLH transcription factor 2 (ASCL2), inhibitor of DNA binding 1 (ID1), and iodothyronine deiodinase 2 (DiO2), have been identified to regulate EVTs' formation and functions.^13^, ^14^, ^15^, ^16^, ^17^ Specifically, transcription factor family activator protein 2 (TFAP2C) is crucial for activating EVT-active genes during the early differentiation of EVTs,^18^ while NOTUM, a negative regulator of WNT signaling, facilitates the transition from trophoblast stem cells (TSCs) to EVTs.^19^ In the maturation phase, EVT cells significantly influence secretory functions through the TGF-β signaling pathway.^20^ Additionally, the transcription factor E74-like factor 3 (ELF3) regulates human leukocyte antigen-C (HLA-C) expression, while NOD-like receptor (NLR) genes modulate immune evasion mechanisms, collectively impacting EVT functions.^21^ These findings underscore the diverse mechanisms regulating EVT differentiation and lay a solid foundation for further research into novel genes that may elucidate EVT's roles. WAS/WASL-interacting protein family member 1 (WIPF1) is a member of the WIP family of proteins that are involved in cytoskeletal organization and cell signaling.^22^ This protein plays a significant role in actin cytoskeleton dynamics, which is crucial for various cellular processes, including cell migration, adhesion, and morphology.^23^ Recent studies have shed light on the multifaceted roles of WIPF1 in various cellular contexts. For instance, multiple studies have demonstrated that WIPF1 is essential for the formation of podosome in migrating cells, emphasizing its role in facilitating cell motility through actin polymerization.^24^, ^25^, ^26^ These findings align with previous work showing that WIPF1 interacts with the Arp2/3 complex, a critical regulator of actin filament branching and network formation.^27^ In addition to its role in cytoskeletal dynamics, WIPF1 has been implicated in the modulation of signaling pathways that govern cellular responses. Recent research indicates that WIPF1 can affect pathways such as the Rho GTPase signaling cascade, which is vital for cytoskeletal rearrangement and cell migration.^28^ The interplay between WIPF1 and these pathways suggests that this protein may also contribute to cellular decision-making processes during events such as immune responses and tissue remodeling. Moreover, WIPF1's involvement extends to disease contexts. Previous studies highlighted its potential role in cancer, where WIPF1 expression levels correlated with increased cell invasion and migration in breast cancer cell lines.^25^^,^^29^ These studies underscore the importance of WIPF1 as a potential therapeutic target for inhibiting tumor progression.

However, the regulatory mechanism of WIPF1 in EVTs' invasion has not yet been clarified. In this study, we aimed to determine the role of WIPF1 in EVT invasion and assess the level of WIPF1 in the placental villi of recurrent spontaneous abortion (RSA) patients. Our findings will contribute to clarifying the mechanism of placentation, understanding the pathogenesis of RSA, and identifying therapeutic targets against RSA.

## Materials and methods

### Informed consent and human sample collection

First-trimester placental villous tissues (6–8 weeks) were obtained from healthy pregnant women (*n* = 10, aged 18–35) who resorted to voluntary termination of pregnancy and from women who had suffered RSA (*n* = 10, aged 18–35). RSA was defined as the occurrence of two or more consecutive first-trimester pregnancy losses of unknown etiology.^30^ The samples were collected via real-time ultrasound-directed aspiration in the First Affiliated Hospital of Chongqing Medical University, immediately frozen in liquid nitrogen for further use, or fixed overnight in 4% paraformaldehyde solution followed by paraffin embedding. All the experimental procedures were consistent with the ethical principles of the Declaration of Helsinki and were approved by the Institutional Animal Care and Use Committee of Chongqing Medical University (IACUC-CQMU) (License number: 2022125). All the samples were collected with written informed consent from the participants.

### Spontaneous abortion-prone mouse model

CBA/J and DBA/2 mice strains (6–8 weeks, 20–22 g) were acquired from HFK Bioscience Co., Ltd., with Bal/b/c mice obtained from Chongqing Medical University Animal Laboratory. The animal experimental procedures were approved by the Ethics Committee of Chongqing Medical University. The mice were fed with standard rodent chow and water at 20 °C–26 °C and kept in a 30%–70% humidified environment at a 12 h/12 h light/dark cycle. Normal pregnancy models were established by randomly mating the female CBA/J and male Bal/b/c mice, while spontaneous abortion-prone mice models were established by randomly mating female CBA/J with male DBA/2 mice.^31^ The presence of a vaginal plug was used as a marker for gestational day 0.5 (GD 0.5). Dams were sacrificed on GD 8.5 via cervical dislocation. Each feto-placental unit was separated from the implantation site via laparotomy. Samples were stored immediately at −80 °C or fixed and embedded in paraffin.

### CTB and EVT isolation and culture

CTBs and EVTs were isolated from human placental villous tissues using established protocols.^32^^,^^33^ To detach the CTBs, we minced and digested the placental villous tissues (6–8 weeks of gestation, *n* = 5) with 0.25% trypsin (Beyotime) three times in a 37 °C water bath. The resultant cell suspension was gently laid on a Percoll™ gradient (70%–10%, in 25% steps) (Cytiva, Uppsala, Sweden). Cells that sedimented at the 30%–40% region of the Percoll™ gradient were collected. For EVT isolation, the placental villous tissues were rinsed in phosphate-buffered saline (PBS), minced, and digested twice with TrypLE™ (Thermo Fisher, NY, USA) in a 37 °C water bath for 20 min. The cell digestion was sequentially filtered with 70 μm and 40 μm cell strainers. Cells were collected and resuspended with DMEM/F-12 media and then gently laid on the surface of Lymphoprep™ (Axis-Shield, Oslo, Norway). The cells between Lymphoprep™ and the cell suspension junction were collected and washed with DMEM/F-12 media. The cells were then cultured on flasks pre-coated with 1 mg/mL fibronectin in PBS at 37 °C and 5% CO_2_ for 45 min. Immunofluorescence was used to assess the purity of the cells.

### Isolation of ITGA6^+^ CTBs and induction into TSCs

ITGA6^+^ CTBs were isolated by immunomagnetic purification using the EasySep PE selection kit (STEMCELL, 17664, Canada) and a PE-conjugated anti-integrin subunit alpha 6 (ITGA6) antibody. Sterile villous tissues from 6-to-8-week normal pregnancies were collected in PBS on ice. Blood contaminants were removed by adding sterile PBS to the sample in a sterile dish. The villous tissues were minced, mixed with TrypLE™ digestion enzyme solution (1:10), and incubated at 37 °C for 30 min with gentle shaking every 2 min. The digested solution was filtered through a 70 μm strainer, and the filtrate was collected into a 50 mL tube containing twice the volume of buffer. Cells were centrifuged at 2500 rpm for 10 min, resuspended in 80 μL buffer, and counted. A 100 μL aliquot of the cell suspension was used. After FcR Blocker (10 μL) and PE-conjugated anti-ITGA6 antibody (0.3 μg) were added, the cells were incubated at room temperature for 15 min. Selection cocktail (10 μL) was added, and incubation continued for another 15 min. 5 μL of RaipidSpheres™ (vortexed for 30 s before use) was added to the mixture, and incubation continued for 10 min. The volume was adjusted to 2.5 mL with PBS containing 2% fetal bovine serum (FBS) and 1 mM EDTA. The FACS tube was placed in a magnet for 5 min at room temperature, and the supernatant was removed. This process was repeated twice. ITGA6-positive CTBs were purified immunomagnetically. The 24-well plate was coated with 0.5 μL/mL iMatrix-511 (Wako, 385–07361, Japan) at 37 °C for 10 min, then seeded with ITGA6-positive CTBs, and incubated in hTSC medium. The culture medium was prepared with the following: advanced DMEM/F-12 (Gibco, 12634010, USA) supplemented with 0.5% penicillin-streptomycin (Beyotime, C0222, Shanghai, China), 0.1 mM 2-mercaptoethanol (Gibco, 21985-023, USA), 0.2% FBS (Gibco, a5669401, USA), 0.3% bovine serum albumin (BSA) (Sigma, A9418, USA), 1% ITS-X supplement (Gibco, 51500056, USA), 1.5 μg/mL l-ascorbic acid (Sigma, A5960, USA), 0.5 μM A83-01 (MCE, HY-10432A, USA), 2 μM CHIR99021 (Selleck, S1263, USA), 1 μM SB431542 (MCE, HY-10431, USA), 50 ng/mL EGF (PeproTech, AF-100-15, USA), 5 μM Y27632 (Selleck, S1049, USA), and 0.8 mM VPA (Sigma, P4543, USA).

### Culture of hTSCs

Each well of the 6-well plate was coated with 0.25 μg/mL of iMatrix-511 and hTSC medium at 37 °C for at least 10 min, and each well was inoculated in it according to 1 × 10^6^ hTSCs, and cultured in an incubator at 37 °C and 5% CO_2_ with 2 mL of hTSC medium, and the medium was changed every day. When the cell density reached 60%–80%, the cells were dissociated with TrypLE™ for 10–15 min at 37 °C and then were passaged into new 6-well plates coated with iMatrix-511 at a 1:20 split ratio.

### Differentiation of hTSCs into EVTs

hTSCs were grown to 80% confluence in the hTSC medium and dissociated with TrypLE™ for 10–15 min at 37 °C. For the differentiation of EVT cells, hTSCs were seeded in a 6-well plate pre-coated with 1 mg/mL Col IV (Sigma, C5533, USA) at a density of 0.75 × 10^5^ cells per well and cultured in 2 mL of EVT medium (advanced DMEM/F-12 supplemented with 0.5% penicillin-streptomycin, 0.1 mM 2-mercaptoethanol, 0.2% FBS, 0.3% BSA, 1% ITS-X supplement, 100 ng/mL neuregulin 1 (NRG1; Novusbio, NBP2-35099, USA), 7.5 mM A83-01, 2.5 mM Y27632, and 4% knockout serum replacement (Thermo Fisher, 10828028, USA)). Matrigel (CORNING, 356234, USA) was added to a final concentration of 2% shortly after suspending the cells in the medium. Cells were harvested on day 3 of differentiation.

### Cell line culture

The EVT cell line, HTR-8/SVneo, which was a gift from Professor Charles. H. Graham^34^ of Queen's University, Canada, was cultured in RPMI 1640 medium (Life Technologies, CA, USA). The choriocarcinoma cell line, JEG-3, gifted by Professor Yan-Ling Wang at the Institute of Zoology, Chinese Academy of Sciences, Beijing, China, was cultured in MEM medium (Life Technologies). HEK293T cells obtained from the American Type Culture Collection (VA, USA) were grown in DMEM/F-12, GlutaMAX medium (Thermo Fisher, MA, USA). All cell lines were supplemented with 10% FBS (Lonsera, Canelones, Uruguay) together with 100 units/mL penicillin and 100 μg/mL streptomycin and maintained in a humidified atmosphere of 21% O_2_ and 5% CO_2_ at 37 °C.

### Human placental villous explant culture

Human first-trimester placental villous tissues (6–8 weeks of gestation, *n* = 3) were obtained and dissected into villous explants with diameters of 3–5 mm. The tissues were cultured following a published protocol.^35^ In brief, the villous explants were anchored onto 6 cm dishes pre-coated with Matrigel (1:19) and cultured in DMEM/F-12 medium (Procell, Wuhan, China) supplemented with 15% FBS in a 37 °C and 5% CO_2_ incubator. By 24 h, the villi had attached to the Matrigel-coated plates. For transient transfection, WIPF1 knockdown plasmid constructs were transfected into explant cultures using Lipofectamine 3000 transfection reagent (Life Technologies, CA, USA). The GFP reporter gene was used to monitor transfection efficiency. Outgrowth of the villous explants was observed at 24 h and 48 h post-transfection. The outgrowth distance was quantified using ImageJ software (NIH, MD, USA). In detail, the outgrowth distance was analyzed by calculating the area formed by the outgrowth perimeter at 48 h (expanded area) and subtracting the area formed by the outgrowth perimeter at 24 h (initial area). The difference was then normalized by dividing by the initial area to obtain a ratio, which was used for statistical analysis. Results were presented as normalized to the control group.

### Analysis of single-cell gene expression signatures of human first-trimester EVTs

The deep learning method, called Deep Embedding for Single-cell Clustering (DESC),^36^ was used to search for key genes that are specifically expressed in the EVTs. The human first-trimester fetal–maternal interface single-cell RNA-sequencing dataset (E-MTAB-6701) was used for trophoblast subtype classification.^37^ In detail, all sample data were analyzed using the annotation provided by the processed data file. Then, R was used to map the data to the UMAP. The data was collected for further analysis in accordance with the annotation by Vento-Tormo, Efremova.^37^ The cell number and subtypes in each cell lineage category were counted and visualized as cell clusters using the t-distribution random neighborhood embedding (t-SNE) plots.^38^ Cell clusters were colored based on gene expression or different features using the gene expression atlas.^39^ Venn diagram was used to show the overlapping genes expressed in each EVT subtype.^40^ Further, biaxial scatter plots and violin plot analysis were performed using the Fluidigm's SINGuLAR™. The Analysis Toolset v3.5.2^41^ was used to show the expression of WIPF1 in different cell types.

### Lentiviral constructions

A lentiviral transduction system was applied to knock down WIPF1 or alpha-actinin 4 (ACTN4) using short hairpin RNA (shRNA) or to overexpress WIPF1 using the full-length open reading frames (ORF) clone.^8^^,^^9^^,^^42^ For ACTN4 or WIPF1 knockdown, pSIH1-based constructs were generated by cloning WIPF1-shRNA (targeting 5′-CCAGAGCCAUAUGUACAAA-3′) or ACTN4-shRNA (targeting 5′- GCCACACTATCGGACATCAAA-3′) by cloning annealed shRNA oligos into the EcoRI/BamHI sites of the pSIH1 plasmid. For WIPF1 single knockdown, two types of pSIH1-based constructs were generated: one with puromycin resistance and GFP, and the other with hygromycin B resistance (without GFP). For ACTN4 single knockdown, the pSIH1-based constructs with hygromycin B resistance (without GFP) were utilized. For the double knockdown of ACTN4 and WIPF1, both ACTN4-shRNA and WIPF1-shRNA were cloned into separate pSIH1 plasmids carrying hygromycin B resistance (without GFP). A negative control used a non-targeting shRNA. The transfections were conducted using polyethyleneimine transfection reagent (Cat#24765, Polysciences, PA, USA). HEK293T cells were transfected with the designed lentiviral constructs along with packaging plasmids (psPAX2 and pVSVG). At 8 h post-transfection, the transfection media were replaced with complete growth media. Media containing lentiviral particles were used to culture HTR-8/SVneo cells. For the double knockdown, ACTN4-shRNA and WIPF1-shRNA were cloned into separate pSIH1 plasmids without GFP expression. HTR-8/SVneo cells were co-infected with both lentiviral constructs, allowing for the concurrent suppression of ACTN4 and WIPF1 expression. Puromycin (10 μg/mL, Beyotime) or hygromycin B (50 μg/mL, Beyotime) selection was utilized for 12 h. Long-term maintenance of the stable transfectants was achieved using media containing 1 μg/mL puromycin or hygromycin B (5 μg/mL). Only the shRNA vector was used as a negative control.

For the WIPF1 overexpression (WIPF1-OE) construct, the coding sequence of WIPF1 was cloned into the EcoRI/BamHI sites of the CD513B1 vector via homologous recombination, following the manufacturer's instructions of the ClonExpress® II One Step Cloning Kit (Vazyme, Nanjing, China). The transfections were conducted with the polyethyleneimine transfection reagent (Transgenic, Shanghai, China). HEK293T cells were transfected with the designed lentiviral constructs and packaged plasmids (psPAX2 and pVSVG). The transfection media were replaced with complete growth media at 8 h post-transfection. The culturing media of the HEK293T cells were collected after 2 days. The cell lines (HTR-8/SVneo, JEG-3) were cultured with fresh media containing lentiviral particles. After 12 h, the media were replaced with complete growth media containing 10 μg/mL of puromycin. However, fresh media containing 1 μg/mL puromycin was used for long-term maintenance of the stable transfecting trophoblast cell lines overexpressing or suppressing WIPF1.

### Quantitative reverse transcription PCR

Total RNA was extracted using the RNAiso Plus kit (Takara, Tokyo, Japan). PrimeScript RT Master Mix (Takara) was used to synthesize the cDNA, after which quantitative reverse transcription PCR was performed with TB Green® Premix Ex Taq™ II (Takara). Primers and cDNA dilutions were done in accordance with a previous protocol.^43^ The primers were synthesized at the Beijing Genomics Institute. Sequences of the primers are listed in Table S1.

### Western blotting

Tissues were weighed and cut into pieces on ice and homogenized in a 60 μL 1 × RIPA lysis buffer (Beyotime, P0013B), containing 50 mM Tris (pH 7.4), 150 mM NaCl, 1% Triton X-100, 1% sodium deoxycholate, and 0.1% SDS, supplemented with 1 mM phenylmethanesulfonyl fluoride (PMSF; Beyotime). The cell suspension was centrifuged at 12,000 *g* at 4 °C for 30 min. The supernatant was carefully collected, and the protein concentration was quantified using the BCA Protein Assay Kit (Beyotime). Total protein lysates were boiled in a 5 × SDS loading buffer (Beyotime) for 10 min. The cell samples were treated with 1 × RIPA lysis buffer and PMSF. Total protein lysates were boiled in a 5 × SDS loading buffer for 10 min and then subjected to SDS-PAGE electrophoresis. Immunoblotting was performed using specific antibodies (Table S2).

### Immunofluorescence staining

Paraffin-embedded tissue sections were antigen-retrieved in EDTA (pH 8.0) (Solarbio, Beijing, China), permeated with 0.3% Triton X-100 solution for 10 min, and the nonspecific antibody binding sites were blocked with 5% BSA. The cells were incubated with the primary antibodies at 4 °C overnight. Secondary fluorescent antibodies were used to probe the cells at 37 °C for 1 h. The nuclei were stained with Hoechst (Thermo Fisher, IL, USA).

For cellular immunofluorescence, primary EVTs were initially cultivated in confocal dishes and transfected with sh-WIPF1 and OE-WIPF1 plasmids using Lipofectamine 3000 for 48 h, followed by PBS rinsing. hTSCs, along with their derived EVTs and HTR-8/SVneo cells, were subjected to lentiviral infection to establish stable interference or overexpression of WIPF1 and stable interference of ACTN4. Subsequently, cells were fixed with ice-cold methanol at −20 °C for 5 min, permeabilized with 0.1% Triton-X-100 for 10 min, and blocked with 3% BSA for 1 h. The cells were incubated with the primary antibodies at 4 °C overnight. Secondary fluorescent antibodies were used to probe the cells at 37 °C for 1 h. The nuclei were stained with Hoechst.

For podosome assessment, cells were fixed with 4% formaldehyde for 10 min, permeabilized with 0.5% Triton-X-100 for 7.5 min, and blocked with 3% BSA for 30 min. Cells were then incubated with mouse anti-cortactin primary antibody at 4 °C overnight, followed by 30 min of incubation with AF647-conjugated goat anti-mouse IgG secondary antibodies (1:200) or TRITC-conjugated phalloidin (1:200, Yeasen, Shanghai, China). To quantify podosome formation, at least 30 cells were randomly selected in each experiment, and the number of cells with podosomes was counted and expressed as a percentage of the total cells examined. Co-localization of F-actin and cortactin, indicating podosome formation, was visualized using confocal microscopy. Images were taken at a set of fluorescence excitation wavelengths (*λ*_ex_ = 405 nm; 488 nm; 555 nm; 640 nm) and within fluorescence emission wavelength ranges (*λ*_em_ = 430–510 nm; 505–550 nm; 600–650 nm; 670–720 nm) using the Nikon C2 confocal microscope (Nikon, Tokyo, Japan). The image and co-localization analysis were done with the Image J software (NIH). The primary antibody dilutions are shown in Table S2.

### Immunohistochemical staining

The tissues were fixed with 4% paraformaldehyde (Beyotime) and embedded following a previous protocol.^44^ In brief, the sections were dewaxed with xylene, rehydrated in gradient ethanol (100%, 95%, 85%, and 75%), and then rinsed with PBS. The sections were exposed to heat-induced epitope retrieval in EDTA (pH 8.0). Endogenous peroxidase activity was stopped with 3% H_2_O_2_. The sections were incubated with a blocking buffer containing 5% *w*/*v* BSA (Solarbio) and 10% goat serum (Beyotime) to prevent non-specific antibody binding. This was done at 37 °C for 30 min. The sections were incubated overnight with primary antibodies (Table S2) at 4 °C. Biotinylated secondary antibodies (ZSGB-BIO, Beijing, China) were used to detect the primary antibodies (Table S2). Following treatment with streptavidin-HRP (ZSGB-BIO), the sections were stained with DAB. The nuclei were counterstained with hematoxylin. Mouse or rabbit IgG was used for negative controls. Images were photographed with an Olympus upright microscope (DP73, Tokyo, Japan) and the Olympus imaging software Ver.5172.

### Transwell invasion assay

The transwell invasion assay was performed following a previous protocol.^45^ HTR-8/SVneo cell suspensions (containing 1 × 10^5^ cells diluted in 200 μL FBS-free medium) were added onto the transwell inserts (8 μm pore size, BD Biosciences, NJ, USA) coated with FBS-free medium-diluted Matrigel (1:6) for 30 min in a 37 °C incubator. Then, the inserts were placed into 24-well plates containing 500 μL medium supplemented with 10% FBS. After incubation for 24 h, the cells that had remained on the upper chambers were removed with a cotton swab, while those that had invaded and gotten trapped in the pores of the lower chamber were fixed with ice-cold methanol for 10 min and stained with crystal violet for 25 min. The stained cells were counted from at least three different fields.

### Scratch assay

Cell migration was determined with the scratch assay following Kaspi's protocol.^46^ HTR-8/SVneo and JEG-3 cells were cultured for 24 h in six-well plates at 37 °C. When cell confluence reached almost 95%, the cell monolayer was scratched with a 200 μL pipette tip. The scratch area was photographed at 0 h and 36 h post-treatment. Image J was used to measure the scratch area. A decrease in the scratch area was an indication of migration. The percentage migration was then calculated.

### Proteomics analysis and co-immunoprecipitation assay

Co-immunoprecipitation was performed using a previous protocol.^47^ In detail, HTR-8/Svneo cells transfected with WIPF1-OE plasmid were seeded on 6 cm dishes for 48 h. The cells were treated with lysis buffer containing a protease inhibitor cocktail of 1 mM dithiothreitol (DTT, Beyotime) and 1 mM PMSF for 30 min. The cell lysates were immediately incubated with rabbit WIPF1 mAb at 4 °C overnight on a rotating mixer. Rabbit or mouse IgG mAb was used as a negative control. The immune complex was precipitated with the protein A/G beads (Bimake, TX, USA) at 4 °C for 2 h in a HulaMixer™ Sample Mixer (Life Technologies). The beads were centrifuged and washed five times with lysis buffer containing protease inhibitor cocktail. The proteins were resolved with 5 × SDS loading buffer or eluted with SDT buffer (2% SDS, 100 mM DTT, 100 mM Tris–HCL, pH 7.6).

Proteomic analysis of WIPF1 interacting proteins, via mass spectrometry, was performed by Aptbiotech Co. (Shanghai, China). Proteins were immunoprecipitated from HTR8/Svneo cells using WIPF1 antibody. Mass spectrometry was performed with the Easy nLC1200/Q Exactive system (Thermo Fisher, CA, USA). The alkylated sample was treated with trypsin (mass ratio 1:50) at 37 °C for 20 h. After salt removal, the enzymatic product was frozen-dried, re-dissolved in 0.1% formic acid solution, and stored at −20 °C. After the chromatographic column was balanced with 95% liquid A (0.1% formic acid aqueous solution), the peptides' mass-charge ratio from the automatic injector to the Trap column was collected using 20 fragment profiles (MS2 scan) after each full scan. Carbamidomethyl (C) was set as a fixed modification. Oxidation (M) was set as a variable modification. A maximal number of missed cleavages was set to 2 with the tryptic enzymatic specificity. Moreover, peptide or fragment mass tolerance was 20 ppm or 0.1 Da. The results were filtered by score ≥20. The RAW files were interpreted using Mascot 2.2 to identify peptides. Furthermore, all generated proteomics data were searched against the Uniprot human proteome reference database (Homo_sapiens_188433_20200217).

The molecular interaction between WIPF1 and ACTN4 was validated via co-immunoprecipitation and immunoblotting, as described above. In brief, proteins immunoprecipitated with the WIPF1 rabbit mAb were detected via immunoblotting using the mouse ACTN4 mAb. The immunoprecipitation efficiencies were verified with the immunoprecipitation antibody. The whole cell lysate without the co-immunoprecipitation procedure was used as the input control.

### Actin polymerization assay

The actin polymerization assay was performed following the manufacturer's instructions (BK003, Cytoskeleton, CO, USA)^48^, ^49^, ^50^ to assess the impact of specific protein silencing on actin polymerization in HTR-8/SVneo cells. HTR-8/SVneo cell lysates, obtained after gene silencing, were employed in each experiment. Cell lysates containing approximately 60–120 μg total protein were mixed with pyrene actin oligomers (0.4 mg/mL) in a 96-well microplate. Polymerization was initiated by adding a 10 × actin polymerization buffer. Fluorescence kinetics were monitored from the top using an Infinite® 200 PRO microplate reader (Tecan, Zurich, Switzerland) to determine the initial rate of actin polymerization. The fluorescence kinetics were recorded under the following conditions: emission wavelength set to a range of 400–440 nm with a step size of 20 nm, excitation wavelength fixed at 340 nm, an integration time of 20 μs, a total of 60 cycles, and a continuous monitoring duration of 60 min. Readings were taken at 1-min intervals, and each emission scan was repeated three times.

### Matrix degradation assay

The matrix degradation assay was performed using previous protocols.^51^, ^52^, ^53^, ^54^ In brief, the coverslips (Nest, CA, USA) were initially treated with 40 μL droplets of a 1:1 mixture of 2 mg/mL Oregon Green® 488 conjugate gelatin (Invitrogen) and 0.1% gelatin (Solarbio) for 10 min. Subsequently, coverslips were washed with PBS and exposed to 4% formaldehyde for 10 min. Following another PBS rinse, coverslips were incubated at 37 °C in RPMI 1640 medium with 15% FBS for 2 h. For cell seeding, 20,000 cells were plated on each coverslip, cultured for 12 h, and then processed for immunofluorescence. Imaging was performed, capturing images from ten fields per sample using a Nikon confocal microscope equipped with a 60 × lens. Subsequently, matrix degradation was analyzed using Image J.

### Molecular docking

The protein structures of WIPF1 (PDB ID: 2A41) and ACTN4 (PDB ID: 6OA6) were retrieved from the Protein Data Bank. Molecular docking was performed using the HDOCK program,^55^, ^56^, ^57^ which employs the Hybrid Docking strategy.^57^ A total of 100 binding conformations were generated, and the conformation with the lowest docking score was selected as the final model. A more negative docking score indicates a more probable binding model. Protein–protein docking was conducted to obtain the complex model, which was then analyzed and visualized using PyMOL software (Version 3.0.3). Docking scores were calculated based on the iterative scoring function ITScorePP or ITScorePR,^58^^,^^59^ with typical docking scores for protein–protein complexes around −200.

### Point mutation

Point mutation was performed using the QuickMutation™ Gene Site-Directed Mutagenesis Kit (Beyotime, D0206S) and the WIPF1-OE overexpression plasmid. The primers for site-directed mutagenesis were diluted to a final concentration of 10 μM each. The PCR reaction mixture was prepared according to the instructions provided in the manual and mixed thoroughly before PCR amplification. After PCR, 1 μL of DpnI was added directly to the PCR reaction mixture and incubated at 37 °C for 5 min. Following DpnI digestion, 10 μL of the DpnI-digested mutagenesis product was added to 100 μL of competent cells for transformation. The point-mutated plasmid was then extracted for subsequent use.

### Statistical analysis

Data were analyzed using GraphPad Prism 7.0 (GraphPad, USA). Each experiment was performed in triplicate. The data were expressed as mean ± standard deviation. The relative mRNA and protein levels, cell migration rate, invasion index, and number of podosomes between groups were analyzed using an independent-samples *t*-test. To account for podosome size differences, podosomes were categorized into small, medium, and large groups based on size.^60^ The watershed algorithm was applied to separate clustered podosomes and prevent misclassification. *p* values < 0.05 were considered statistically significant.

## Results

### The DESC method identified WIPF1 in the EVTs of the human placenta

**Figure 1:**
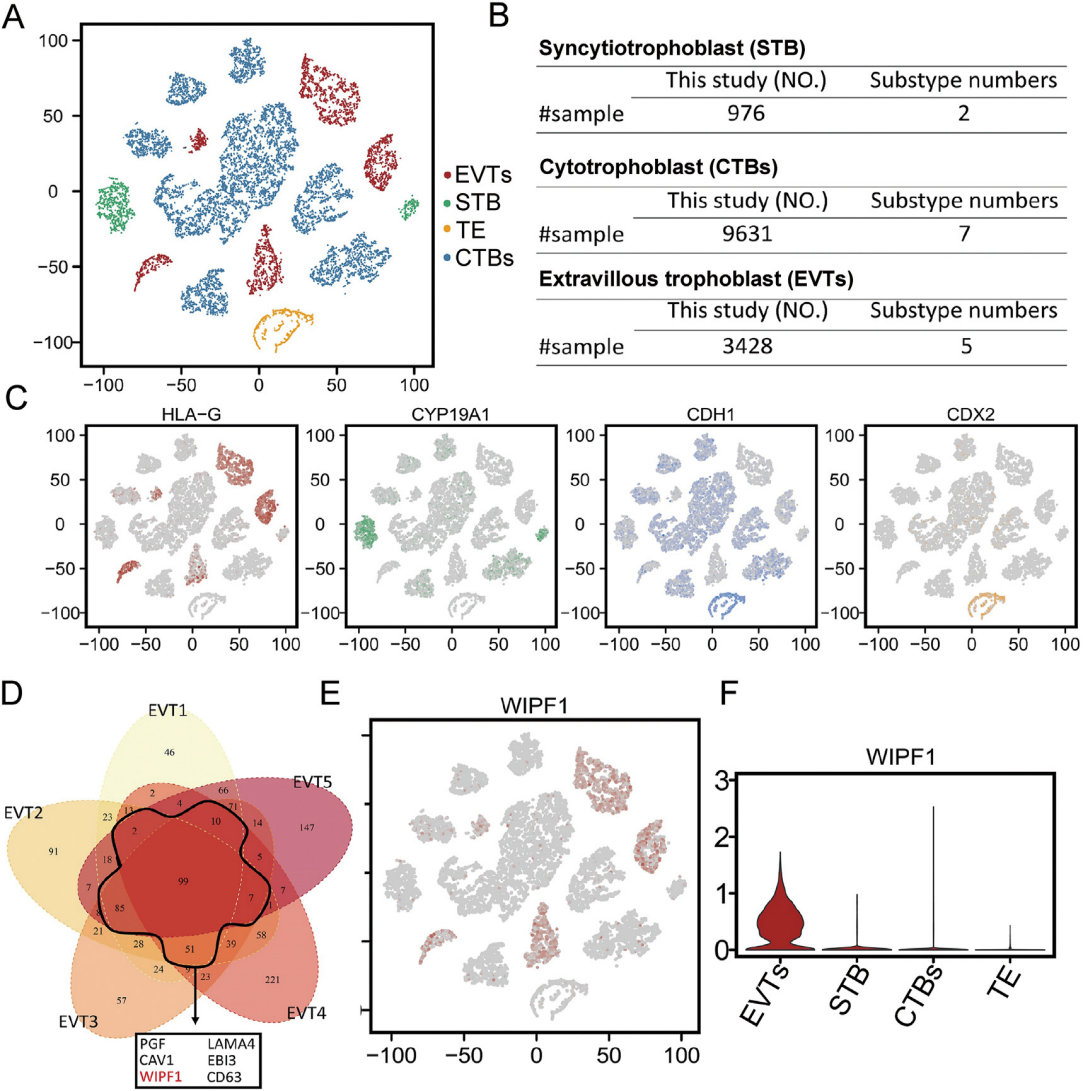
Identification of major trophoblast subtypes at the maternal–fetal interface and differentially expressed genes (DEGs) via the deep learning method called deep embedding for single-cell clustering (DESC) method. **(A)** t-distribution random neighborhood embedding (t-SNE) plots from human placental single-cell RNA sequencing data showing the various trophoblast lineages and the trophoblastic ectoderm (TE). **(B)** The number of subtypes of each trophoblast lineage. **(C)** Annotation of the DESC method and the expression distribution of known marker genes for each lineage or cell type (HLA-G, EVT marker; CYP19A1, STB marker; CDH1, villous CTB marker; CDX2, TE marker). **(D)** The Venn diagram illustrating the genes that are expressed in each of the five EVT subtypes in the gene set maps. **(E)** Biaxial scatter plots of WIPF1 expression patterns in different trophoblast lineages and TE. **(F)** The violin plots showing the expression of WIPF1 in the four major trophoblast lineages. CTB, cytotrophoblast; EVT, extravillous trophoblast; STB, syncytiotrophoblast; HLA-G, human leukocyte antigen-G; CYP19A1, cytochrome P450 family 19 subfamily A member 1; CDH1, cadherin 1; CDX2, caudal type homeobox 2; WIPF1, WAS/WASL interacting protein family member 1.

The published single-cell RNA-sequencing data of the human early feto–maternal interface were applied to explore novel differentially expressed genes involved in EVT functioning.^37^ The cell clustering and gene expression patterns among each cell type were analyzed using an iterative self-learning method, DESC.^36^ Three major trophoblast lineages (EVTs, syncytiotrophoblasts, villous cytotrophoblasts) and trophectoderm cells were colored and visualized in the t-SNE plot (Fig. 1A). Each lineage or cell type was further divided into several clusters, representing the cell heterogeneity of each lineage (Fig. 1A). The number of cells or subtypes of each lineage is listed in Figure 1B. Using known marker genes (HLA-G, CYP19A1, CDH1, or CDX2), we could classify the sample that resembles EVTs, syncytiotrophoblasts, villous CTBs, or trophectoderm cells, respectively. The region distribution labelled with known marker genes of each cluster expression is presented in Figure 1C. The differentially expressed genes in five EVT subtypes were obtained via Venn analysis, showing the expression of a number of genes among more than four EVT subtypes. These genes^61^, ^62^, ^63^, ^64^, ^65^ (Table S4) included placental growth factor (PGF), caveolin 1 (CAV1), laminin subunit alpha 4 (LAMA4), Epstein–Barr virus induced 3 (EBI3), and tetraspanin-30 (CD63), which have been reported to participate in regulating EVT functioning (Fig. 1D). As a novel differentially expressed gene among different trophoblast lineages, WIPF1 was found to be expressed in almost all EVT subtypes (Fig. 1E). Moreover, violin plots of WIPF1 expression showed that WIPF1-positive cells tended to be enriched in EVTs than in trophectoderm cells and the other major trophoblast lineages (Fig. 1F). These data suggest that WIPF1 might be a novel gene regulating human EVT's functions.

### WIPF1 is exclusively expressed in the EVTs of the human placenta

**Figure 2:**
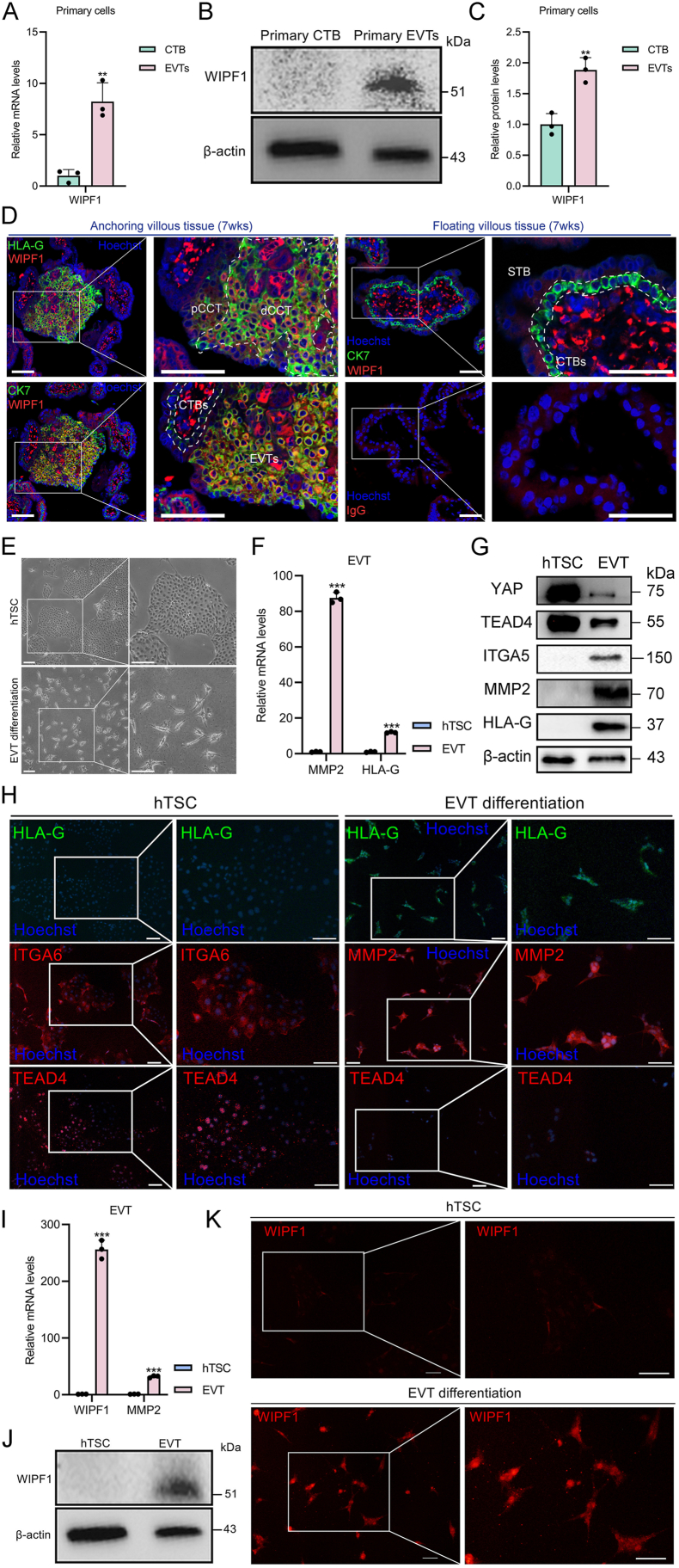
Analysis of WIPF1 expression in placental trophoblasts and human trophoblast stem cell (hTSC)-derived extravillous trophoblast (EVT) differentiation. **(A)** mRNA levels of WIPF1 in human primary cytotrophoblasts (CTBs) and EVTs in the first-trimester placental villi, relative to the mRNA levels of GADPH. ∗∗*p* < 0.01. **(B)** Protein levels of WIPF1 in human primary CTBs and EVTs in the first-trimester placental villi. **(C)** Quantified protein levels of WIPF1 in CTBs and EVTs, relative to the protein levels of β-actin. ∗∗*p* < 0.01. **(D)** Immunofluorescence images of the expression of WIPF1 (red), cytokeratin 7 (CK7; green), and human leukocyte antigen-G (HLA-G; green) in human first-trimester placental villi. The illustration demonstrates the expression of WIPF1 in anchoring or floating first-trimester (6–8 weeks, *n* = 3) placental villi. STB, syncytiotrophoblast; dCCT, distal cell column trophoblast; pCCT, proximal cell column trophoblast. Non-specific staining verification with immunoglobulin G (IgG; red) was taken as a control. The blue colors represent nuclei stained with Hoechst. Scale bar, 100 μm. **(E)** Bright-field images of hTSCs and differentiated EVTs. Scale bars, 200 μm. **(F)** mRNA expression levels of EVT markers, including HLA-G and matrix metallopeptidase 2 (MMP2), in hTSCs and EVTs, relative to the mRNA levels of GADPH. ∗∗∗*p* < 0.001. **(G)** Protein levels of hTSC markers, including Yes-associated Protein (YAP) and TEA domain transcription factor 4 (TEAD4), as well as EVT markers, including integrin subunit alpha 5 (ITGA5), MMP2, and HLA-G, were examined in hTSC and EVT samples. **(H)** Immunofluorescence images of the expression of TEAD4 (red), HLA-G (green), and MMP2 (red) in hTSC and EVT differentiation. The blue colors represent nuclei stained with Hoechst. Scale bars, 25 μm. **(I)** mRNA levels of WIPF1 and MMP2 in hTSCs and EVTs, relative to the mRNA levels of GADPH. ∗∗∗*p* < 0.001. **(J)** Protein levels of WIPF1 in hTSCs and EVTs. **(K)** Immunofluorescence images of the expression of WIPF1 in hTSC and EVT differentiation. Scale bars, 25 μm. WIPF1, WAS/WASL interacting protein family member 1.

Consistent with the DESC analysis, the mRNA (Fig. 2A) and protein (Fig. 2B, C) levels of WIPF1 were found to be higher in invasive primary EVTs compared with primary CTBs. Likewise, we found that WIPF1 was localized to the distal cell column trophoblast, which is the only EVT lineage in the first-trimester placental anchoring villi (Fig. 2D). In contrast, no or lower WIPF1 protein was localized to CTBs and syncytiotrophoblasts in the first-trimester placental floating villi and anchoring villi (Fig. 2D). A weak fluorescence was obtained with the negative control antibodies (anti-IgG primary antibody) (Fig. 2D).

We further established an *in vitro* differentiation model by inducing invasive EVT differentiation from hTSCs isolated from early pregnancy villous tissue. The successfully differentiated EVTs exhibited a mesenchymal-like morphology (Fig. 2E), with a significant up-regulation of EVT markers and a concurrent down-regulation of hTSC markers (Fig. 2F–H), indicating efficient EVT induction. Notably, WIPF1 expression was markedly up-regulated following EVT induction from hTSCs (Fig. 2I–K). These findings suggest that WIPF1 is exclusively expressed in the EVTs of the human placenta.

### WIPF1 promotes EVT migration, invasion, and differentiation

**Figure 3:**
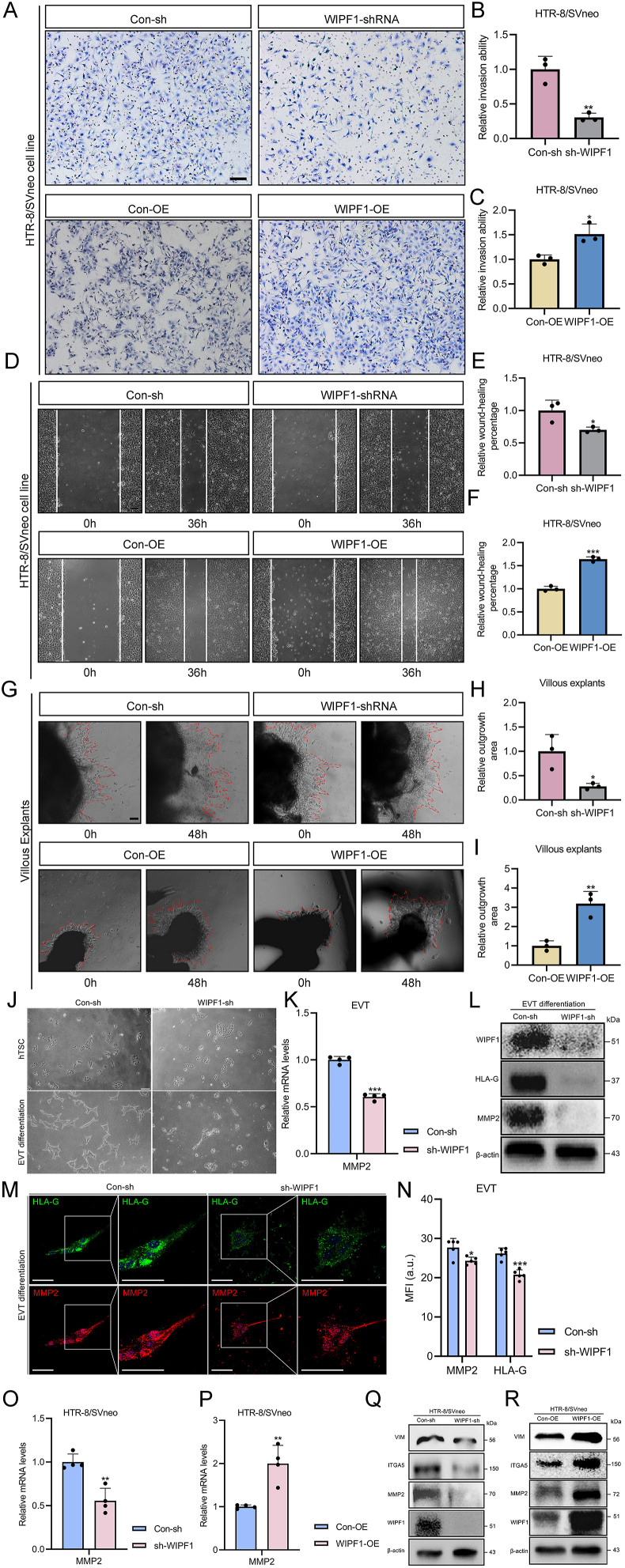
WIPF1 promotes trophoblast migration, invasion, and extravillous trophoblast (EVT) differentiation *in vitro*. **(A)** The invasive abilities of the sh-WIPF1 HTR-8/SVneo cells and WIPF1-OE HTR-8/SVneo cells after 24 h of treatment (*n* = 3). **(B)** Differences in the invasive abilities of the sh-WIPF1 HTR-8/SVneo cells after 24 h of treatment (*n* = 3). ∗∗*p* < 0.01. **(C)** Differences in the invasive abilities of the WIPF1-OE HTR-8/SVneo cells after 24 h of treatment (*n* = 3). ∗*p* < 0.05. **(D)** The migration abilities of sh-WIPF1 HTR-8/SVneo cells and WIPF1-OE HTR-8/SVneo cells after 36 h of treatment (*n* = 3). **(E)** Differences in the migration abilities of sh-WIPF1 HTR-8/SVneo cells after 24 h of treatment (*n* = 3). ∗∗*p* < 0.01. **(F)** Differences in the migration abilities of WIPF1-OE HTR-8/SVneo cells after 24 h of treatment (*n* = 3). ∗∗*p* < 0.01. **(G)** The outgrowth areas of the sh-WIPF1 placental villous explants and the WIPF1-OE placental villous explants at 24 h and 48 h post-transfection. **(H)** The relative outgrowth areas of the sh-WIPF1 villous explants after 24 h and 48 h of treatment. ∗∗*p* < 0.01. **(I)** The relative outgrowth areas of the WIPF1-OE villous explants after 24 h and 48 h of treatment. ∗∗∗*p* < 0.001. **(J)** Bright-field images of EVT differentiation with Con-sh control or WIPF1 knockdown via lentiviral interference. Scale bar, 200 μm. **(K)** mRNA levels of matrix metallopeptidase 2 (MMP2) in sh-WIPF1 EVTs, relative to the mRNA levels of GADPH. ∗∗∗*p* < 0.001. **(L)** Protein levels of WIPF1 and EVT markers in Con-sh and WIPF1-sh cells in EVT differentiation. **(M)** Immunofluorescence images of the expression of human leukocyte antigen-G (HLA-G; green) and MMP2 (red) in sh-WIPF1 EVT differentiation. The blue colors represent nuclei stained with Hoechst. Scale bars, 25 μm. **(N)** Statistical plot of mean fluorescence intensity (MFI) of HLA-G and MMP2 in sh-WIPF1 EVT differentiation. au is the unit of average fluorescence intensity. ∗*p* < 0.05 and ∗∗∗*p* < 0.001. **(O, P)** mRNA levels of MMP2 in sh-WIPF1 and WIPF1-OE HTR-8/SVneo cells, relative to the mRNA levels of GADPH. ∗∗*p* < 0.01. **(Q, R)** Protein levels of invasion markers in WIPF1-sh and WIPF1-OE HTR-8/SVneo cells. WIPF1, WAS/WASL interacting protein family member 1.

WIPF1 knockdown and overexpression in HTR-8/SVneo cells were achieved using a lentiviral system, with efficiencies confirmed by western blotting and quantitative reverse transcription PCR, showing significant reduction or increase in WIPF1 expression, respectively (Fig. S1B, C and Fig. 3Q, R). WIPF1 knockdown significantly reduced the invasiveness of HTR-8/SVneo cells by approximately 3.3-fold, while WIPF1 overexpression increased their invasiveness by approximately 1.5-fold compared with that of the control cells (Fig. 3A–C). Also, the knockdown significantly reduced the migration of the cells by 1.4-fold, while the overexpression increased their migration by 1.6-fold compared with that of the control cells (Fig. 3D–F). These were similarly observed in the JEG-3 cell line (Fig. S2). Moreover, the knockdown reduced the outgrowth of the villous explants, while the overexpression increased their outgrowth significantly (Fig. 3G–I).

To further investigate the impact of WIPF1 on EVT differentiation, we employed an hTSC-induced EVT model using lentiviral-mediated knockdown. Our results revealed that WIPF1 knockdown significantly impaired the morphological differentiation of EVTs (Fig. 3J), and the expression of key EVT markers was markedly down-regulated compared with controls (Fig. 3K–N). Consistently, in the HTR-8/SVneo cell line, WIPF1 knockdown led to the down-regulation of pro-invasion markers, including vimentin (VIM), integrin subunit alpha 5 (ITGA5), and matrix metallopeptidase 2 (MMP2), while its overexpression resulted in their up-regulation (Fig. 3O–R). These findings suggest that WIPF1 promotes trophoblast cell migration and invasion by up-regulating key pro-invasion genes.

### WIPF1 regulates podosome formation and invasion through interaction with ACTN4

**Figure 4:**
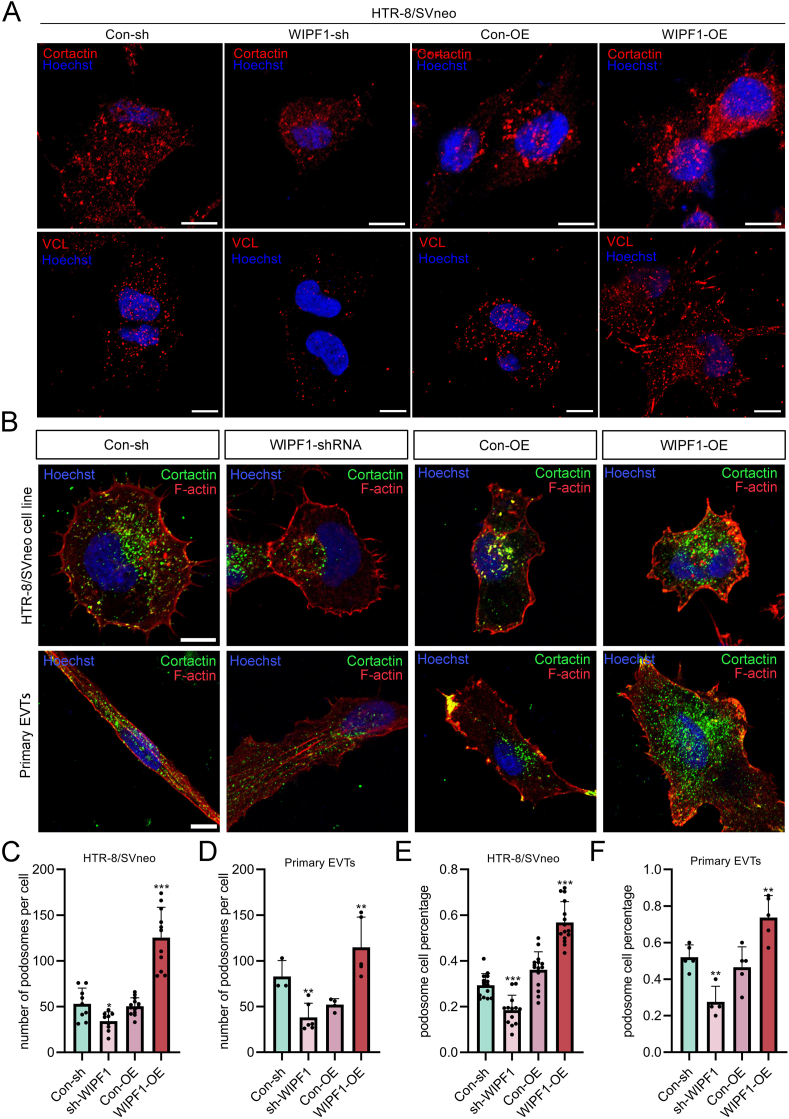
WIPF1 promotes podosome formation by targeting and up-regulating podosome-related proteins. **(A)** Immunofluorescence images of cortactin (red) or vinculin (VCL; red) in WIPF1-sh and WIPF1-OE HTR-8/SVneo cells. Scale bar, 10 μm. **(B)** Immunofluorescence images of cortactin and phalloidin in WIPF1-sh HTR-8/SVneo cells and WIPF1-OE HTR-8/SVneo cells or primary extravillous trophoblasts (EVTs). The fluorescent dots (yellow) in the cytoplasm represent the podosomes. Phalloidin, red. Cortactin, green. **(C, D)** The number of podosomes in the cytoplasm of sh-WIPF1 HTR-8/SVneo cells and WIPF1-OE HTR-8/SVneo cells (*n* = 30 cells) and sh-WIPF1 primary EVTs and WIPF1-OE primary EVTs (*n* = 30 cells). ∗*p* < 0.05, ∗∗*p* < 0.01, and ∗∗∗*p* < 0.001. **(E, F)** The percentage of podosome cells relative to the total cell population of sh-WIPF1 HTR-8/SVneo cells (*n* = 50 cells), WIPF1-OE HTR-8/SVneo cells (*n* = 50 cells), sh-WIPF1 primary EVTs (*n* = 50 cells), and WIPF1-OE primary EVTs (*n* = 50 cells). ∗∗*p* < 0.01 and ∗∗∗*p* < 0.001. WIPF1, WAS/WASL interacting protein family member 1.

Cortactin, a key regulator of podosome formation, was up-regulated upon WIPF1 overexpression in HTR-8/SVneo cells (Fig. 4A), while its expression was reduced following WIPF1 knockdown (Fig. 4A). Additionally, vinculin, a cell–matrix contact protein used to determine late-podosome localization, was up-regulated following WIPF1 overexpression (Fig. 4A), while its expression was down-regulated after WIPF1 knockdown. Immunofluorescence intensity analysis revealed significant differences compared with the control group (Fig. S3). In addition, we observed that talin 1 (TLN1), one of the podosome-associated proteins, was colocalized with WIPF1 within the cytoplasm (Fig. S4). When the podosomes were visualized by the co-localization of F-actin and cortactin in the HTR-8/SVneo cell line and primary EVTs, we found that the number of podosomes in the WIPF1-shRNA cells was significantly lower than that in the control cells. On the contrary, the number of podosomes in the WIPF1-OE cells was considerably higher than that in the control cells (Fig. 4B–D). Subsequently, we determined the percentage of cells forming podosomes and then measured the podosome count per cell in each treatment group. In the WIPF1-OE group, podosome formation was significantly increased compared with the OE-control group, while the sh-WIPF1 group displayed a marked decrease compared with the sh-control group (Fig. 4E, F). These findings highlight the pivotal role of WIPF1 in regulating podosome formation processes in HTR-8/SVneo cells and primary EVTs.

**Figure 5:**
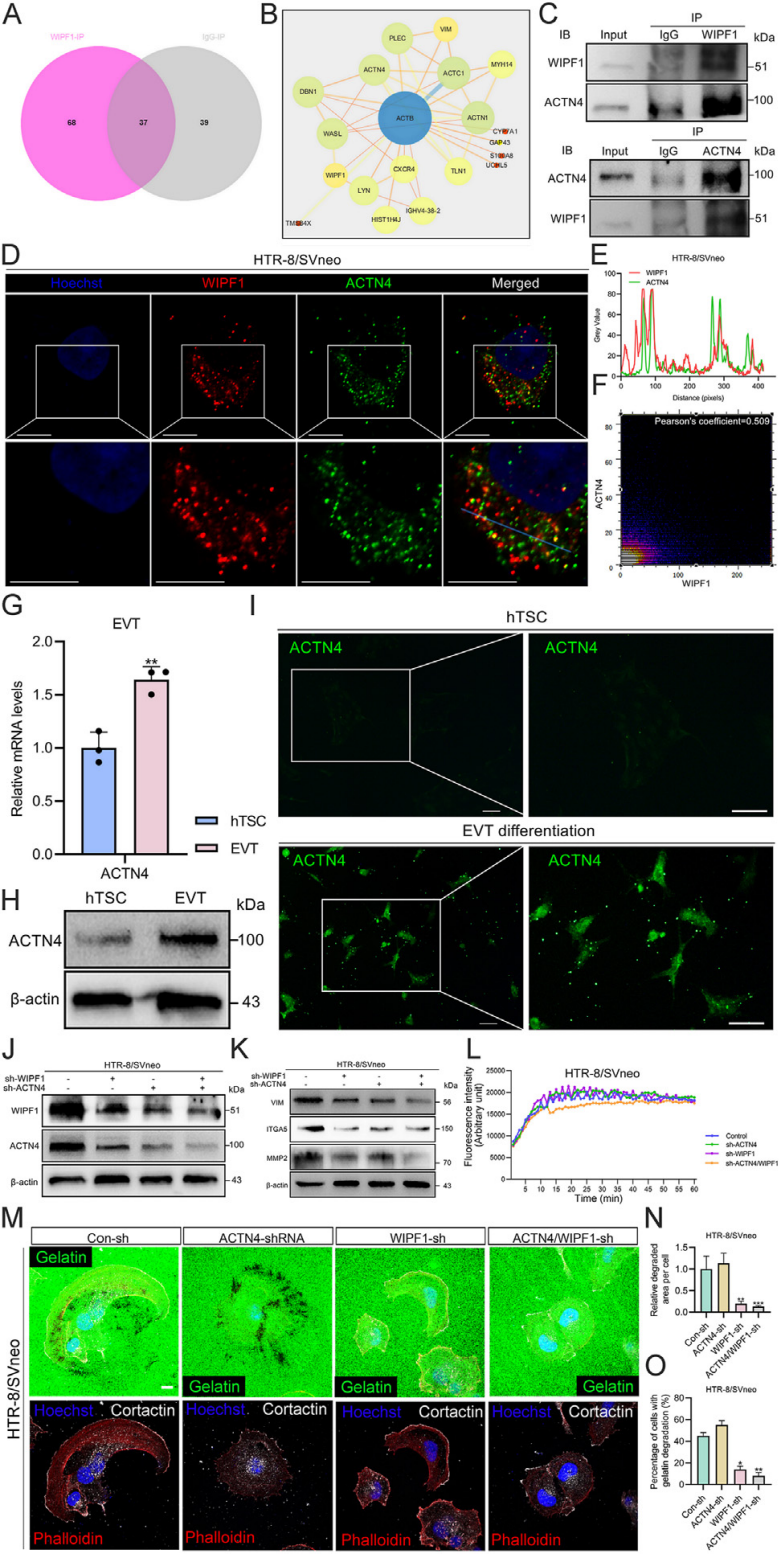
WIPF1-ACTN4 interaction and its role in podosome function during extravillous trophoblast (EVT) invasion. **(A)** Venn analysis of the WIPF1 interactome identified in the WIPF1-OE HTR-8/SVneo cells. The light red and grey represent anti-WIPF1 and anti-IgG immunoprecipitates, respectively. The dark red represents the non-specific interacting protein of the WIPF1 immunoprecipitates. **(B)** Protein–protein interaction network via STRING. Nodes, circles. Edges, lines. **(C)** WIPF1 and ACTN4 immunoprecipitates stained with ACTN4 and WIPF1 in WIPF1-OE HTR8/SVneo cells. **(D**–**F)** Immunofluorescence images of the expression of ACTN4 (green) and WIPF1 (red) in HTR-8/SVneo cells. The blue colors represent nuclei stained with Hoechst. Scale bars, 10 μm. Colocalization analysis was performed, and Pearson's correlation coefficient was calculated to assess their interaction. A gray value analysis plot of fluorescence intensity along the crossed-out distance is shown. **(G)** mRNA levels of ACTN4 in human trophoblast stem cells (hTSCs) and EVTs, relative to the mRNA levels of GADPH. ∗∗*p* < 0.01. **(H)** Protein levels of ACTN4 in hTSCs and EVTs. **(I)** Immunofluorescence images of the expression of ACTN4 in hTSC and EVT differentiation. Scale bars, 25 μm. **(J)** Protein levels of WIPF1 and ACTN4 after single and double knockdown of WIPF1 and ACTN4 in HTR-8/SVneo cells. **(K)** Protein levels of invasion markers after single and double knockdown of WIPF1 and ACTN4 in HTR-8/SVneo cells. **(L)** Actin polymerization assay was conducted using HTR-8/SVneo cells stably transfected with lentiviral vectors containing shRNA constructs targeting WIPF1 (WIPF1 knockdown), ACTN4 (ACTN4 knockdown), WIPF1 and ACTN4 (WIPF1/ACTN4 double knockdown), and vector control groups. The assay monitored the kinetics of pyrene actin polymerization in 60 min. The experiment was performed in triplicate. **(M)** Immunofluorescence images for F-actin (red), Oregon Green® 488 conjugate gelatin (green), cortactin (white), and nuclei (blue) in HTR-8/SVneo cells with WIPF1 and/or ACTN4 knockdown following incubation for 12 h on the matrix. The black areas mark areas of podosome-mediated degradation, which appear beneath the cells. Scale bars, 10 μm. **(N)** Quantitative analysis of the relative area of gelatin degradation zones associated with podosomes for different treatment groups. *n* = 30 fields; ∗∗*p* < 0.01 and ∗∗∗*p* < 0.001. **(O)** Quantitative evaluation of the proportion of cells containing podosome-associated gelatin degradation area for different treatment groups. *n* = 30 fields; ∗*p* < 0.05 and ∗∗*p* < 0.01. ACTN4, alpha-actinin 4; WIPF1, WAS/WASL interacting protein family member 1.

Moreover, we identified 105 and 76 proteins from the WIPF1-OE cell proteins and IgG immunoprecipitated proteins, respectively. Through Venn analysis, we selected 68 WIPF1-interacting proteins for further studies (Fig. 5A and Table S3). Based on the String, we selected the immunoprecipitated proteins that interacted with WIPF1 (Table S3). Subsequently, we established the protein–protein interaction network of WIPF1-interacting proteins. We found the hub genes (such as ACTN4, PLEC, and TLN1) which may regulate actin cytoskeleton and podosome formation by interacting with WIPF1 (Fig. 5B). Among those proteins, ACTN4 was identified to interact with WIPF1 (Fig. 5C). Also, WIPF1 and ACTN4 were found to partially co-localize in HTR-8/SVneo cells, suggesting a potential interaction between the two proteins (Fig. 5D–F). Additionally, ACTN4 expression was significantly up-regulated during EVT differentiation induced by hTSCs (Fig. 3G–I). Besides, WIPF1-interacting factors were commonly expressed in the actin cytoskeleton, cytosol, focal adhesion, *etc*. (Fig. S5A). Further analysis of the molecular functional annotation showed that WIPF1 interactors were enriched in vinculin binding, structural constituent of the cytoskeleton, actin filament binding, *etc*. (Fig. S5B). Also, via the gene ontology analysis, we found that WIPF1 interaction factors were enriched with actin polymerization or depolymerization, actin filament organization, actin filament-based movement, and other biological processes (Fig. S5C). A Kyoto Encyclopedia of Genes and Genomes (KEGG) pathway enrichment analysis was performed with 68 proteins (Fig. S5D), showing actin cytoskeleton and metabolic pathways, which are essential drivers of cell migration and invasion.

We further conducted individual or dual knockdown experiments of WIPF1 and ACTN4 in HTR-8/SVneo cells. The results showed that the expression levels of WIPF1 and ACTN4 were down-regulated to those observed in the individual knockdown groups after dual knockdown of ACTN4/WIPF1 (Fig. 5J; Fig. S6). This suggests that the WIPF1/ACTN4 complex was inhibited, accompanied by damage to the invasion markers, indicating that WIPF1 and ACTN4 jointly participate in maintaining the cell invasion capability (Fig. 5K). Notably, individual knockdown of either WIPF1 or ACTN4 in HTR-8/SVneo cells did not significantly change actin polymerization kinetics. However, simultaneous knockdown of WIPF1/ACTN4 showed a significant reduction in actin polymerization capability (Fig. 5L). Furthermore, we conducted gelatin degradation assays to assess the impact of WIPF1 single knockdown or WIPF1/ACTN4 simultaneous knockdown on podosome-mediated extracellular matrix degradation. In these assays, gelatin degradation is visualized as dark areas beneath the cells, reflecting the activity of podosomes. Our results revealed a significant reduction in gelatin degradation, indicating impaired extracellular matrix degradation, when WIPF1 was knocked down alone or in combination with ACTN4 (Fig. 5M−O). These results suggest a potential interactive role of WIPF1 and ACTN4 in modulating actin polymerization during podosome formation and podosome-mediated matrix degradation of trophoblast cell invasion.

**Figure 6:**
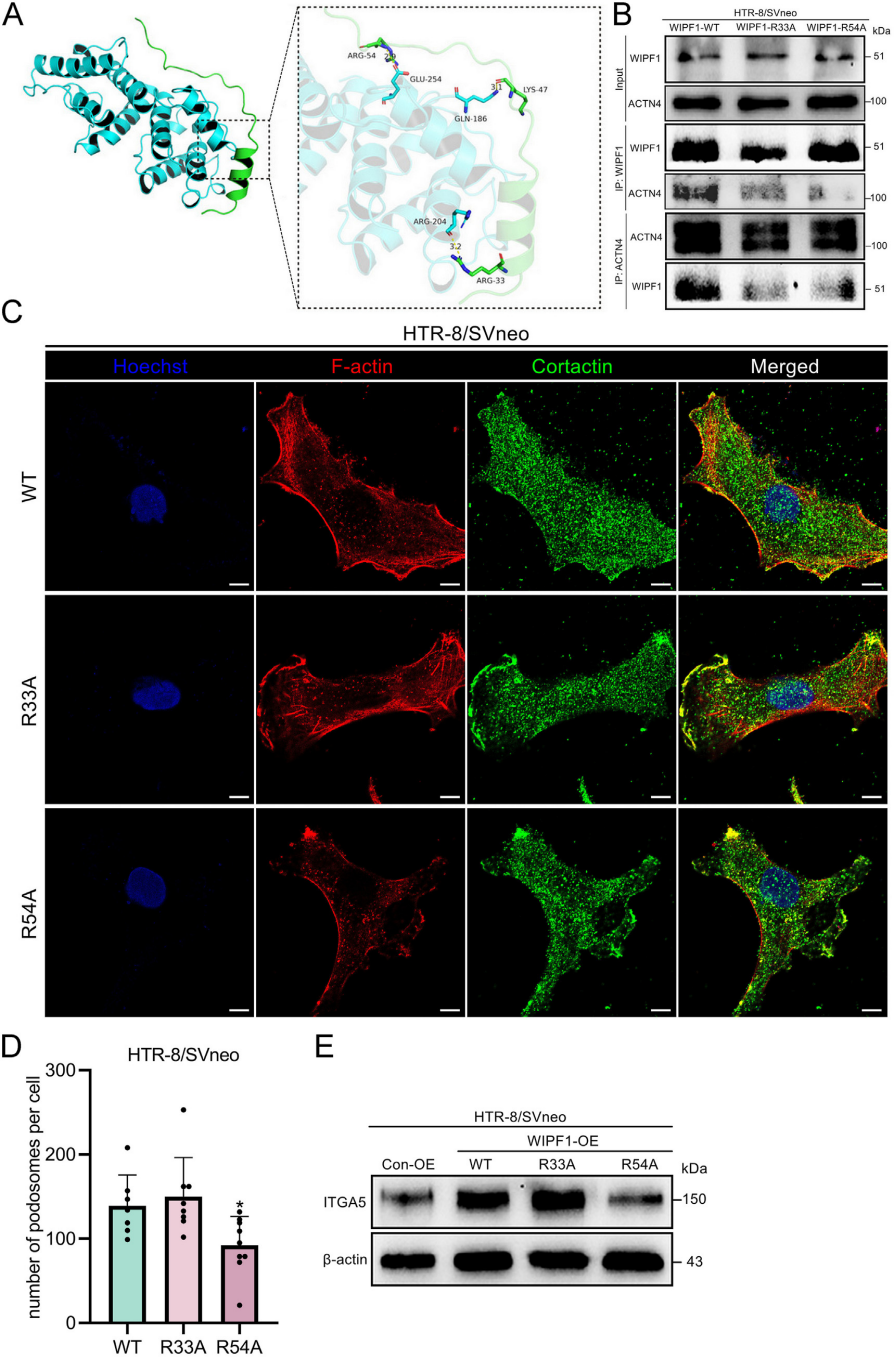
Disruption of WIPF1/ACTN4 interaction affects podosome formation and invasion markers in HTR-8/SVneo cells. **(A)** Protein–protein interaction diagram and names of amino acid residues in the part of the protein–protein interaction that generates hydrogen bonds, where the cyan part is the ACTN4 protein and the green part is the WIPF1 protein. Hydrogen bonds are indicated by yellow dashed lines. **(B)** WIPF1 and ACTN4 immunoprecipitates in WT, R33A, and R54A WIPF1-overexpressing HTR-8/SVneo cells. **(C)** Immunofluorescence staining images of F-actin (red) and cortactin (green) in WT, R33A, and R54A WIPF1-overexpressing HTR-8/SVneo cells. Scale bars, 10 μm. **(D)** The number of podosomes in the WT, R33A, and R54A WIPF1-overexpressing HTR-8/SVneo cells. ∗*p* < 0.05. **(E)** Protein levels of invasion marker integrin subunit alpha 5 (ITGA5) in the WT, R33A, and R54A WIPF1-overexpressing HTR-8/SVneo cells. ACTN4, alpha-actinin 4; WIPF1, WAS/WASL interacting protein family member 1.

We further explored the molecular mechanism underlying the interaction between WIPF1 and ACTN4. Through molecular docking simulations, we predicted the binding of WIPF1 and ACTN4 proteins, with a protein–protein docking score of −202.81 (data not shown). In addition, we identified specific amino acid sites where WIPF1 interacted with ACTN4 via hydrogen bonds, including ARG33, ARG54, and LYS47 (Fig. 6A). To further validate this interaction, we performed point mutations on WIPF1 and used co-immunoprecipitation experiments. Results showed that mutations at the ARG33 and ARG54 sites (R33A and R54A) significantly disrupted the interaction between WIPF1 and ACTN4 (Fig. 6B). Further, the WIPF1-R54A mutation significantly impaired podosome formation (Fig. 6C, D) and led to a down-regulation of the invasion marker ITGA5 (Fig. 6E). This indicates that the R54A mutation disrupts the WIPF1-ACTN4 interaction, highlighting that the WIPF1-ACTN4 interaction, particularly at the R54A site, plays a critical role in regulating podosome function and EVT invasion.

### Decreased WIPF1 expression in the placental villi is involved in RSA

**Figure 7:**
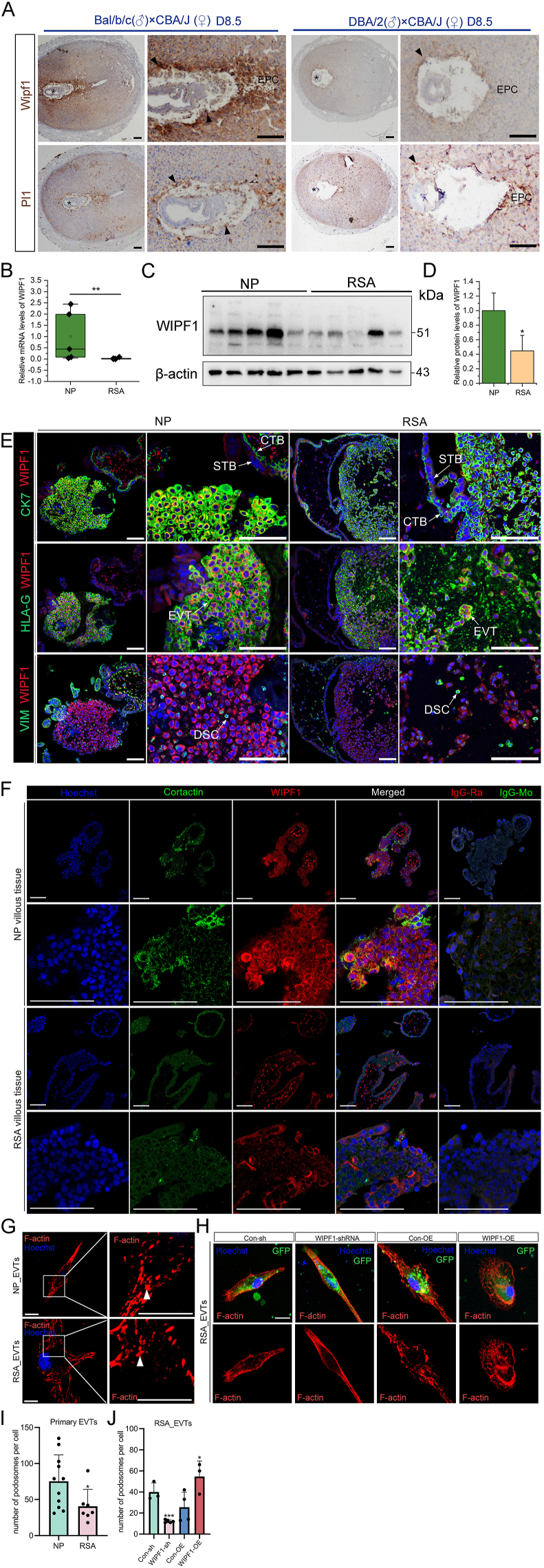
The expression of WIPF1 in the early maternal–fetal interface of humans or mice with recurrent spontaneous abortion (RSA) and their respective controls. **(A)** Immunohistochemical images of WIPF1 expression at the mouse maternal–fetal interface on pregnancy day 8.5 (PD8.5). EPC, ectoplacental cone. Scale bar, 200 μm. The black arrows indicate trophoblast giant cells (TGCs). The asterisks indicate the site of embryo attachment. **(B)** mRNA levels of WIPF1 in healthy human first-trimester placental villi and RSA placental villi, relative to the mRNA levels of β-actin. ∗∗*p* < 0.01. **(C)** Protein levels of WIPF1 in human first-trimester placental villi and RSA placental villi, relative to the protein levels of β-actin. **(D)** Quantified protein levels of WIPF1, relative to the protein levels of β-actin. ∗*p* < 0.05. **(E)** Immunofluorescence images of the expression of WIPF1 (red), cytokeratin 7 (CK7; green), and human leukocyte antigen-G (HLA-G; green) in human first-trimester placental villi and RSA placental villi. The blue colors represent nuclei stained with Hoechst. Scale bar, 100 μm. The white arrows represent maternal decidual cells. Scale bar, 100 μm. **(F)** Immunofluorescence images of the expression of cortactin (green, IgG-Mouse) and WIPF1 (red, IgG-Rabbit) in normal pregnancy (NP) or RSA first-trimester placental villous tissues. The blue colors represent nuclei stained with Hoechst. Scale bar, 100 μm. **(G)** Images of primary extravillous trophoblasts (EVTs) from NP and RSA patients' villous tissue, as stained with phalloidin (for F-actin). The fluorescent dots within the cytoplasm represent the podosomes. Phalloidin, red. Scale bars, 10 μm. **(H)** Primary EVTs from RSA patients' villous tissue expressing human WIPF1-OE or sh-WIPF1, together with the control constructs, as stained with phalloidin (for F-actin). The fluorescent dots within the cytoplasm represent the podosomes. Phalloidin, red. GFP, green. Scale bars, 10 μm. **(I)** The number of podosomes in the NP or RSA EVTs. ∗*p* < 0.05. **(J)** The number of podosomes in the WIPF1-OE EVTs, sh-WIPF1 RSA EVTs, and their respective controls (containing GFP expression). ∗*p* < 0.05 and ∗∗∗*p* < 0.001. WIPF1, WAS/WASL interacting protein family member 1.

We observed a decreased WIPF1 expression in the placental cone and trophoblast giant cells on pregnancy day 8.5 (PD8.5) in the RSA mouse compared with those from the healthy controls (Fig. 7A). Also, the mRNA and protein levels of WIPF1 were significantly lower in the human RSA villous tissues than in the control villous tissues (Fig. 7B–E). Hence, the down-regulation of WIPF1 in the first-trimester placental villi is associated with RSA. Moreover, we found that WIPF1 was co-localized with cortactin in the healthy first-trimester EVTs, but cortactin was down-regulated in the RSA first-trimester EVTs (Fig. 7F; Fig. S7), which suggests that WIPF1 may play a role in podosome formation in human EVTs. We observed a significantly decreased number of podosomes in RSA EVTs compared with normal pregnancy EVTs (Fig. 7G–I). This difference was mainly due to a reduction in small-sized podosomes in RSA EVTs (Fig. S8). Also, we observed a significant decrease in podosome number in the WIPF1-shRNA cells compared with the control cells. In contrast, the podosome number in the WIPF1-OE cells was increased compared with that in the control cells (Fig. 7H–J). These results suggest that the down-regulation of WIPF1 in the first-trimester placental villi may contribute to the pathogenesis of RSA.

## Discussion

Understanding the molecular mechanisms underlying trophoblast differentiation is crucial for improving therapeutic strategies for RSA. In this study, we validated the reliability of DESC using it to identify a number of genes (such as PGF, CAV1, LAMA4, EBI3, ID1, DiO2, and CD63) which have been reported to be expressed in the EVTs and hence control their functions.^17^^,^^66^ Also, through DESC and histological analysis, we found that WIPF1 was expressed in the EVTs. It has been revealed that WIPF1 is essential for actin polymerization, which is critical for the formation of several actin-rich subcellular structures, such as podosomes, filopodia, and lamellipodia, during cell invasion.^67^ Deficiency in WIPF1 binding site on the Wiskott-Aldrich syndrome protein (WASL) or in WIPF1 itself caused Wiskott-Aldrich syndrome (WAS).^68^ Aberrant expression of WIPF1 facilitated the invasiveness of several malignancies, such as breast cancer, colorectal cancer, and glioma, by enhancing podosome formation.^69^ Since the migration and invasion of EVTs into the decidua share similarities with the invasion of host tissues by cancer cells,^70^ it could be hypothesized that WIPF1 could promote EVT migration and invasion.

Podosomes, known to be crucially involved in cell migration and invasion,^71^ are significantly influenced by the collaborative actions of WIPF1 and ACTN4 (Fig. 5). Their dynamic interaction likely up-regulates podosome-related proteins,^72^ impacting podosome morphology and function. In testing the above hypothesis, our study uncovers the intricate interplay between WIPF1 and ACTN4 shaping podosome structure and function, as shown in Figure 5. Notably, WIPF1 co-localizes with cortactin (a podosome marker protein) (Fig. 7F) and regulates podosome formation in trophoblast cells through its interaction with ACTN4, a protein we previously identified as a key regulator of actin cytoskeleton remodeling in EVTs. Our findings on ACTN4 are consistent with previous studies,^73^ which also highlight its crucial role in podosome formation and invasion in trophoblast cells. In our hTSC differentiation experiments, we observed a significant up-regulation of ACTN4 expression during EVT differentiation (Fig. 5G–I), highlighting its regulatory role in this process. ACTN4 and WIPF1 are both pro-invasive factors in EVTs, and we observed their individual effects on invasion. Both contribute to podosome formation, with ACTN4 playing a key role in extracellular matrix degradation mediated by podosomes in breast cancer cells.^74^ However, in our experiments, knockdown of ACTN4 alone did not significantly impair matrix degradation in trophoblast cells. This discrepancy may be due to differences between cancer cells and trophoblast cells, or it could suggest that WIPF1 may independently regulate podosome-mediated matrix degradation. Nevertheless, the irreplaceable role of the WIPF1-ACTN4 complex lies in their combined contribution to actin polymerization and the proper formation of podosome structures (Figure 5, Figure 6C). Besides, WIPF1 knockdown resulted in a decrease in ACTN4 expression in both hTSC-induced EVTs and HTR-8/SVneo cells, while overexpression of WIPF1 increased ACTN4 levels (Fig. S9). This is consistent with literature suggesting that WIPF1 promotes epithelial–mesenchymal transition and down-regulates E-cadherin, reducing its competitive interaction with ACTN4.^75^^,^^76^ This alleviates the inhibition on ACTN4, thereby enhancing its effects and increasing cellular invasiveness. This WIP-regulated mechanism may offer new insights into the regulation of trophoblast cell invasion, with potential implications for understanding pregnancy-related complications. Additionally, WIPF1 interacts with actin-related proteins like WASL and ACTN1 (Fig. 5B), highlighting its role in actin dynamics. WASL promotes actin polymerization and the formation of cellular protrusions, while ACTN1 stabilizes actin filaments and enhances the mechanical strength of the cytoskeleton.^77^ Together, their interaction with WIPF1 may facilitate lamellipodia formation, significantly impacting the migration and invasion capabilities of EVTs.

Focal adhesions are crucial structures for cell adhesion, migration, and invasion.^78^ In our study, we found that WIPF1 overexpression significantly increased the adhesion protein vinculin (Fig. 4A). Additionally, WIPF1 overexpression increased ITGA5 expression (Fig. 3R). This suggests that WIPF1 may enhance trophoblast cell invasion by modulating vinculin and ITGA5 levels. Existing literature indicates that vinculin plays a vital role in cell migration and invasion, closely linked to cell adhesion and motility.^79^ Notably, research has shown that ITGA5 co-localizes with vinculin in EVTs, indicating their collaborative role in the invasion process.^80^ Our findings demonstrate that WIPF1 also promotes the expression of other adhesion-related proteins, such as TLN1 and VIM, further supporting WIPF1's involvement in regulating the adhesion complex (Fig. 5B). We propose that WIPF1 modulates intracellular signaling pathways to influence adhesion-related proteins like vinculin and ITGA5, thus facilitating the invasion process.

Also, we observed that WIPF1 promoted the migration and invasion of EVTs by increasing the expression of VIM, ITGA5, and MMP2. Building on these findings, we further explored their effects on matrix degradation (Fig. 5M), thereby deepening our understanding of their roles in cell migration and invasion. Our results indicate that the knockdown of WIPF1, whether alone or in combination with ACTN4, significantly affects the matrix degradation ability of EVTs, while the control group and the knockdown of ACTN4 alone do not show any notable changes. Existing studies have pointed out that MMP2 is crucial for degrading type IV and type V collagen, which is essential for cell migration and invasion.^81^^,^^82^ Thus, WIPF1 may regulate MMP2 activity, influencing matrix remodeling.

It is well known that abnormal pregnancy outcomes, including RSA, preeclampsia, and intrauterine growth restriction, are preceded by shallow EVT invasion.^83^ In this study, we found that WIPF1 was down-regulated in the RSA placental villi of both mice and humans, indicating that WIPF1 may play conservative roles in mice and humans. A previous study by us indicates that the down-regulation of VIM is involved in the pathogenesis of RSA.^17^ Hence, with the knockdown of WIPF1 reducing VIM expression in the present study, we deduce that the down-regulation of WIPF1 in the first-trimester placental villi reduces VIM expression to inhibit EVT invasion and consequently trigger RSA. Also, we found that WIPF1 up-regulates MMP2 (Fig. 3R), a key regulator of trophoblast invasion, which is essential for proper placental development. Insufficient EVT invasion is a hallmark of RSA, and WIPF1 down-regulation is associated with reduced MMP2 expression, which may impair trophoblast invasion and contribute to RSA pathogenesis.^84^ Additionally, as AKT signaling promotes invasion via MMP2 but is diminished in RSA,^85^ and WIPF1 can regulate PI3K/AKT signaling in tumors.^86^ It may modulate trophoblast invasion through a similar mechanism. These findings suggest that WIPF1 supports EVT invasion via the WIPF1-MMP2 axis, and its down-regulation may be involved in RSA. These findings suggest that WIPF1 plays an important role in maintaining EVT invasion and that its down-regulation may contribute to RSA pathogenesis. Further investigation is needed to explore the potential biological significance and clinical implications of these observations.

## CRediT authorship contribution statement

**Cong Li:** Writing – original draft, Visualization, Validation, Data curation. **Shengya Wang:** Formal analysis. **Jing Tang:** Visualization, Validation, Funding acquisition. **Xin Luo:** Validation, Funding acquisition. **Luxing Ge:** Investigation. **Youlong Xie:** Investigation. **Lijuan Fu:** Investigation. **Lingling Ruan:** Investigation. **Enoch Appiah Adu-Gyamfi:** Writing – review & editing. **Fangfang Li:** Supervision, Investigation. **Yingxiong Wang:** Project administration, Funding acquisition, Conceptualization. **Hongbo Qi:** Conceptualization. **Yubin Ding:** Writing – review & editing, Writing – original draft, Supervision, Project administration, Funding acquisition, Conceptualization.

## Ethics declaration

All the experimental procedures were consistent with the ethical principles of the Declaration of Helsinki and were approved by the Institutional Animal Care and Use Committee of Chongqing Medical University (License number: 2022125).

## Funding

This work was funded by the 10.13039/501100001809National Natural Science Foundation of China (No. 82171664, 82271707), the Scientific and Technological Research Program of Chongqing Municipal Education Commission (China) (No. KJZD-K202200408), and Joint Innovation and Development Fund of the Chongqing Natural Science Foundation (CSTB2022NSCQ-LZX0062).

## Conflict of interests

The authors declared no competing interests.

## References

[1] Turco M.Y., Gardner L., Kay R.G. (2018). Trophoblast organoids as a model for maternal-fetal interactions during human placentation. Nature.

[2] Gauster M., Moser G., Wernitznig S., Kupper N., Huppertz B. (2022). Early human trophoblast development: from morphology to function. Cell Mol Life Sci.

[3] Abbas Y., Turco M.Y., Burton G.J., Moffett A. (2020). Investigation of human trophoblast invasion *In vitro*. Hum Reprod Update.

[4] Horvat Mercnik M., Schliefsteiner C., Sanchez-Duffhues G., Wadsack C. (2024). TGFβ signalling: a nexus between inflammation, placental health and preeclampsia throughout pregnancy. Hum Reprod Update.

[5] Dietrich B., Haider S., Meinhardt G., Pollheimer J., Knöfler M. (2022). WNT and NOTCH signaling in human trophoblast development and differentiation. Cell Mol Life Sci.

[6] Ferreira L.M.R., Meissner T.B., Tilburgs T., Strominger J.L. (2017). HLA-G: at the interface of maternal-fetal tolerance. Trends Immunol.

[7] Moser G., Windsperger K., Pollheimer J., de Sousa Lopes S.C., Huppertz B. (2018). Human trophoblast invasion: new and unexpected routes and functions. Histochem Cell Biol.

[8] Zhang Y., Ruan L.L., Li M.R. (2024). Palmitic acid impairs human and mouse placental function by inhibiting trophoblast autophagy through induction of acyl-coenzyme A-binding protein (ACBP) upregulation. Hum Reprod.

[9] Li M.R., Chen E.X., Li Z.H. (2024). HMGB1 regulates autophagy of placental trophoblast through ERK signaling pathway. Biol Reprod.

[10] Chen E.X., Hu S.C., Xu J.Q. (2024). Suppression of GATA3 promotes epithelial-mesenchymal transition and simultaneous cellular senescence in human extravillous trophoblasts. Biochim Biophys Acta Mol Cell Res.

[11] Du L., Deng W., Zeng S. (2021). Single-cell transcriptome analysis reveals defective decidua stromal niche attributes to recurrent spontaneous abortion. Cell Prolif.

[12] Arutyunyan A., Roberts K., Troulé K. (2023). Spatial multiomics map of trophoblast development in early pregnancy. Nature.

[13] Varberg K.M., Iqbal K., Muto M. (2021). ASCL2 reciprocally controls key trophoblast lineage decisions during hemochorial placenta development. Proc. Natl. Acad. Sci. U.S.A.

[14] Cheng J.C., Chang H.M., Leung P.C.K. (2013). Transforming growth factor-β1 inhibits trophoblast cell invasion by inducing snail-mediated down-regulation of vascular endothelial-cadherin protein. J Biol Chem.

[15] Pereira de Sousa F.L., Chaiwangyen W., Morales-Prieto D.M. (2017). Involvement of STAT1 in proliferation and invasiveness of trophoblastic cells. Reprod Biol.

[16] Renaud S.J., Chakraborty D., Mason C.W., Karim Rumi M.A., Vivian J.L., Soares M.J. (2015). OVO-like 1 regulates progenitor cell fate in human trophoblast development. Proc. Natl. Acad. Sci. U.S.A.

[17] Adu-Gyamfi E.A., Lamptey J., Chen X.M. (2021). Iodothyronine deiodinase 2 (DiO_2_) regulates trophoblast cell line cycle, invasion and apoptosis; and its downregulation is associated with early recurrent miscarriage. Placenta.

[18] Kim M., Jang Y.J., Lee M. (2024). The transcriptional regulatory network modulating human trophoblast stem cells to extravillous trophoblast differentiation. Nat Commun.

[19] Shukla V., Moreno-Irusta A., Varberg K.M. (2024). NOTUM-mediated WNT silencing drives extravillous trophoblast cell lineage development. Proc. Natl. Acad. Sci. U.S.A..

[20] Haider S., Lackner A.I., Dietrich B. (2022). Transforming growth factor-β signaling governs the differentiation program of extravillous trophoblasts in the developing human placenta. Proc Natl Acad Sci U S A.

[21] Gu B., Le G.H., Herrera S., Blair S.J., Meissner T.B., Strominger J.L. (2024). HLA-C expression in extravillous trophoblasts is determined by an ELF3-NLRP2/NLRP7 regulatory axis. Proc. Natl. Acad. Sci. U.S.A.

[22] Ramesh N., Antón I.M., Hartwig J.H., Geha R.S. (1997). WIP, a protein associated with wiskott-Aldrich syndrome protein, induces actin polymerization and redistribution in lymphoid cells. Proc Natl Acad Sci U S A.

[23] García E., Machesky L.M., Jones G.E., Antón I.M. (2014). WIP is necessary for matrix invasion by breast cancer cells. Eur J Cell Biol.

[24] Tsuboi S. (2007). Requirement for a complex of Wiskott-Aldrich syndrome protein (WASP) with WASP interacting protein in podosome formation in macrophages. J Immunol.

[25] García E., Ragazzini C., Yu X. (2016). WIP and WICH/WIRE co-ordinately control invadopodium formation and maturation in human breast cancer cell invasion. Sci Rep.

[26] Chou H.C., Antón I.M., Holt M.R. (2006). WIP regulates the stability and localization of WASP to podosomes in migrating dendritic cells. Curr Biol.

[27] Ho H.H., Rohatgi R., Lebensohn A.M. (2004). *Toca*-1 mediates Cdc42-dependent actin nucleation by activating the N-WASP-WIP complex. Cell.

[28] Moreau V., Tatin F., Varon C., Génot E. (2003). Actin can reorganize into podosomes in aortic endothelial cells, a process controlled by Cdc42 and RhoA. Mol Cell Biol.

[29] Escoll M., Gargini R., Cuadrado A., Anton I.M., Wandosell F. (2017). Mutant p53 oncogenic functions in cancer stem cells are regulated by WIP through YAP/TAZ. Oncogene.

[30] Gasbarrini A., Torre E.S., Trivellini C., De Carolis S., Caruso A., Gasbarrini G. (2000). Recurrent spontaneous abortion and intrauterine fetal growth retardation as symptoms of coeliac disease. Lancet.

[31] Clark D.A., McDermott M.R., Szewczuk M.R. (1980). Impairment of host-versus-graft reaction in pregnant mice. II. Selective suppression of cytotoxic T-cell generation correlates with soluble suppressor activity and with successful allogeneic pregnancy. Cell Immunol.

[32] Xie L., Mouillet J.F., Chu T. (2014). C19MC microRNAs regulate the migration of human trophoblasts. Endocrinology.

[33] Nelson D.M., Johnson R.D., Smith S.D., Anteby E.Y., Sadovsky Y. (1999). Hypoxia limits differentiation and up-regulates expression and activity of prostaglandin H synthase 2 in cultured trophoblast from term human placenta. Am J Obstet Gynecol.

[34] Graham C.H., Hawley T.S., Hawley R.G. (1993). Establishment and characterization of first trimester human trophoblast cells with extended lifespan. Exp Cell Res.

[35] Bauer S., Pollheimer J., Hartmann J., Husslein P., Aplin J.D., Knöfler M. (2004). Tumor necrosis factor-alpha inhibits trophoblast migration through elevation of plasminogen activator inhibitor-1 in first-trimester villous explant cultures. J Clin Endocrinol Metab.

[36] Li X., Wang K., Lyu Y. (2020). Deep learning enables accurate clustering with batch effect removal in single-cell RNA-seq analysis. Nat Commun.

[37] Vento-Tormo R., Efremova M., Botting R.A. (2018). Single-cell reconstruction of the early maternal-fetal interface in humans. Nature.

[38] Butler A., Hoffman P., Smibert P., Papalexi E., Satija R. (2018). Integrating single-cell transcriptomic data across different conditions, technologies, and species. Nat Biotechnol.

[39] Papatheodorou I., Moreno P., Manning J. (2020). Expression Atlas update: from tissues to single cells. Nucleic Acids Res.

[40] Jia A., Xu L., Wang Y. (2021). Venn diagrams in bioinformatics. Brief Bioinform.

[41] Gong H., Wang X., Liu B. (2017). Single-cell protein-mRNA correlation analysis enabled by multiplexed dual-analyte co-detection. Sci Rep.

[42] Xie Y., Cao H., Zhang Z., Zhang S., Wang H. (2019). Molecular network of miR-1343 regulates the pluripotency of porcine pluripotent stem cells via repressing OTX2 expression. RNA Biol.

[43] Wu J., He C., Bu J. (2020). Betaine attenuates LPS-induced downregulation of Occludin and Claudin-1 and restores intestinal barrier function. BMC Vet Res.

[44] Duan F.M., Fu L.J., Wang Y.H. (2020). THBS1 regulates trophoblast fusion through a CD36-dependent inhibition of cAMP, and its upregulation participates in preeclampsia. Genes Dis.

[45] Duzyj C.M., Barnea E.R., Li M., Huang S.J., Krikun G., Paidas M.J. (2010). Preimplantation factor promotes first trimester trophoblast invasion. Am J Obstet Gynecol.

[46] Kaspi E., Guillet B., Piercecchi-Marti M.D. (2013). Identification of soluble CD146 as a regulator of trophoblast migration: potential role in placental vascular development. Angiogenesis.

[47] Sun L., Zheng J., Wang Q. (2016). NHERF1 regulates actin cytoskeleton organization through modulation of α-actinin-4 stability. FASEB J.

[48] Li L., Mao B., Yan M. (2019). Planar cell polarity protein dishevelled 3 (Dvl3) regulates ectoplasmic specialization (ES) dynamics in the testis through changes in cytoskeletal organization. Cell Death Dis.

[49] Wen Q., Li N., Xiao X. (2018). Actin nucleator Spire 1 is a regulator of ectoplasmic specialization in the testis. Cell Death Dis.

[50] Li N., Mruk D.D., Wong C.K.C., Lee W.M., Han D., Cheng C.Y. (2015). Actin-bundling protein plastin 3 is a regulator of ectoplasmic specialization dynamics during spermatogenesis in the rat testis. FASEB J.

[51] Sala K., Raimondi A., Tonoli D., Tacchetti C., de Curtis I. (2018). Identification of a membrane-less compartment regulating invadosome function and motility. Sci Rep.

[52] El Azzouzi K., Wiesner C., Linder S. (2016). Metalloproteinase MT1-MMP islets act as memory devices for podosome reemergence. J Cell Biol.

[53] Murphy D.A., Courtneidge S.A. (2011). The 'ins' and 'outs' of podosomes and invadopodia: characteristics, formation and function. Nat Rev Mol Cell Biol.

[54] Riedl J., Crevenna A.H., Kessenbrock K. (2008). Lifeact: a versatile marker to visualize F-actin. Nat Methods.

[55] Yan Y., Tao H., He J., Huang S.Y. (2020). The HDOCK server for integrated protein-protein docking. Nat Protoc.

[56] Yan Y., Zhang D., Zhou P., Li B., Huang S.Y. (2017). HDOCK: a web server for protein-protein and protein-DNA/RNA docking based on a hybrid strategy. Nucleic Acids Res.

[57] Yan Y., Wen Z., Wang X., Huang S.Y. (2017). Addressing recent docking challenges: a hybrid strategy to integrate template-based and free protein-protein docking. Proteins.

[58] Huang S.Y., Zou X. (2008). An iterative knowledge-based scoring function for protein-protein recognition. Proteins.

[59] Huang S.Y., Zou X. (2014). A knowledge-based scoring function for protein-RNA interactions derived from a statistical mechanics-based iterative method. Nucleic Acids Res.

[60] Cao F., Zhou Y., Liu X., Yu C.H. (2020). Podosome formation promotes plasma membrane invagination and integrin-β3 endocytosis on a viscous RGD-membrane. Commun Biol.

[61] Salomon C., Yee S., Scholz-Romero K. (2014). Extravillous trophoblast cells-derived exosomes promote vascular smooth muscle cell migration. Front Pharmacol.

[62] Devergne O., Coulomb-L’Herminé A., Capel F., Moussa M., Capron F. (2001). Expression of Epstein-Barr virus-induced gene 3, an interleukin-12 p40-related molecule, throughout human pregnancy: involvement of syncytiotrophoblasts and extravillous trophoblasts. Am J Pathol.

[63] Ji Y., Zhou L., Wang G., Qiao Y., Tian Y., Feng Y. (2019). Role of LAMA4 gene in regulating extravillous trophoblasts in pathogenesis of preeclampsia. Med Sci Monit.

[64] Reppetti J., Reca A., Seyahian E.A. (2020). Intact caveolae are required for proper extravillous trophoblast migration and differentiation. J Cell Physiol.

[65] Athanassiades A., Lala P.K. (1998). Role of placenta growth factor (PIGF) in human extravillous trophoblast proliferation, migration and invasiveness. Placenta.

[66] Zhao H.J., Klausen C., Zhu H., Chang H.M., Li Y., Leung P.C.K. (2020). Bone morphogenetic protein 2 promotes human trophoblast cell invasion and endothelial-like tube formation through ID1-mediated upregulation of IGF binding protein-3. FASEB J.

[67] Fried S., Matalon O., Noy E., Barda-Saad M. (2014). WIP: more than a WASp-interacting protein. J Leukoc Biol.

[68] Derry J.M., Ochs H.D., Francke U. (1994). Isolation of a novel gene mutated in Wiskott-Aldrich syndrome. Cell.

[69] García E., Jones G.E., Machesky L.M., Antón I.M. (2012). WIP: WASP-interacting proteins at invadopodia and podosomes. Eur J Cell Biol.

[70] Wallace A.E., Fraser R., Cartwright J.E. (2012). Extravillous trophoblast and decidual natural killer cells: a remodelling partnership. Hum Reprod Update.

[71] Redondo-Muñoz J., Escobar-Díaz E., Samaniego R., Terol M.J., García-Marco J.A., García-Pardo A. (2006). MMP-9 in B-cell chronic lymphocytic leukemia is up-regulated by alpha4beta1 integrin or CXCR4 engagement via distinct signaling pathways, localizes to podosomes, and is involved in cell invasion and migration. Blood.

[72] Kinley A.W., Weed S.A., Weaver A.M. (2003). Cortactin interacts with WIP in regulating Arp2/3 activation and membrane protrusion. Curr Biol.

[73] Peng W., Tong C., Li L. (2019). Trophoblastic proliferation and invasion regulated by ACTN4 is impaired in early onset preeclampsia. FASEB J.

[74] Yamaguchi H., Ito Y., Miura N. (2017). Actinin-1 and actinin-4 play essential but distinct roles in invadopodia formation by carcinoma cells. Eur J Cell Biol.

[75] Salvi A., Thanabalu T. (2017). WIP promotes *in-vitro* invasion ability, anchorage independent growth and EMT progression of A549 lung adenocarcinoma cells by regulating RhoA levels. Biochem Biophys Res Commun.

[76] Hayashida Y., Honda K., Idogawa M. (2005). E-cadherin regulates the association between beta-catenin and actinin-4. Cancer Res.

[77] Hamill K.J., Hiroyasu S., Colburn Z.T. (2015). Alpha actinin-1 regulates cell-matrix adhesion organization in keratinocytes: consequences for skin cell motility. J Invest Dermatol.

[78] Carragher N.O., Frame M.C. (2004). Focal adhesion and actin dynamics: a place where kinases and proteases meet to promote invasion. Trends Cell Biol.

[79] Rubashkin M.G., Cassereau L., Bainer R. (2014). Force engages vinculin and promotes tumor progression by enhancing PI3K activation of phosphatidylinositol (3, 4, 5)-triphosphate. Cancer Res.

[80] Kabir-Salmani M., Shiokawa S., Akimoto Y. (2003). Alphavbeta3 integrin signaling pathway is involved in insulin-like growth factor I-stimulated human extravillous trophoblast cell migration. Endocrinology.

[81] Hornebeck W., Emonard H., Monboisse J.C., Bellon G. (2002). Matrix-directed regulation of pericellular proteolysis and tumor progression. Semin Cancer Biol.

[82] Tanaka N., Sakamoto T. (2023). MT1-MMP as a key regulator of metastasis. Cells.

[83] Schatz F., Guzeloglu-Kayisli O., Arlier S., Kayisli U.A., Lockwood C.J. (2016). The role of decidual cells in uterine hemostasis, menstruation, inflammation, adverse pregnancy outcomes, and abnormal uterine bleeding. Hum Reprod Update.

[84] Yan Y., Fang L., Li Y. (2021). Association of *MMP2* and MMP9 gene polymorphisms with the recurrent spontaneous abortion: a meta-analysis. Gene.

[85] Luan X., Li S., Zhao J. (2020). Down-regulation of CCR7 via the AKT pathway and GATA2 inactivation suppressed trophoblast migration and invasion in recurrent spontaneous abortion. Biol Reprod.

[86] Su F., Xiao R., Chen R. (2023). WIPF1 promotes gastric cancer progression by regulating PI3K/Akt signaling in a myocardin-dependent manner. iScience.

