# Supplementary material for: Unraveling the role of the WIPF1/ACTN4 complex in podosome formation of human placental EVTs: Insights into recurrent spontaneous abortion
Source: Genes Dis. 2025 May 2;12(6):101665. doi: 10.1016/j.gendis.2025.101665 (PMC12357052; doi:10.1016/j.gendis.2025.101665)
Supplement: Multimedia component 1 [file mmc1.docx]

# Supplementary Table S1

Sequences of primers for RT-qPCR, lentiviral vector construction, and point mutation.

| Gene & Species | Primer Sequence (5–3′) |
| --- | --- |
| WIPF1 (homo) | F: ACGGCCAACAGGGATAATGAT |
|  | R: GGTTTCGCAGATGTGGATCTT |
| GAPDH (homo) | F: GGAGCGAGATCCCTCCAAAAT |
|  | R: GGCTGTTGTCATACTTCTCATGG |
| CD63 (homo) | F: CAGTGGTCATCATCGCAGTG |
|  | R: ATCGAAGCAGTGTGGTTGTTT |
| EBI3 (homo) | F: TCATTGCCACGTACAGGCTC |
|  | R: GGGTCGGGCTTGATGATGTG |
| LAMA4 (homo) | F: CCACACTCGTCCTTCTCTCTC |
|  | R: AGTTTCCGAACTGACCTAGCC |
| PGF (homo) | F: GAACGGCTCGTCAGAGGTG |
|  | R: ACAGTGCAGATTCTCATCGCC |
| CAV1 (homo) | F: GCGACCCTAAACACCTCAAC |
|  | R: ATGCCGTCAAAACTGTGTGTC |
| HLA-G (homo) | F: GTGGATGATTGGCTGCGAC |
|  | R: TCGTTCAGGGCGAGGTAATC |
| MMP2 (homo) | F: TACAGGATCATTGGCTACACACC |
|  | R: GGTCACATCGCTCCAGACT |
| ACTN4 (homo) | F: TGGAGGTCATATCAGGGGAGC |
|  | R: GAGACCAGCTTGACGCCTTT |
| WIPF1-ECOR1-2/F | ATTCTAGAGCTAGCGAATTCATGCCTGTCCCTCCCCCTC |
| WIPF1-BAMH1-2/R | ATCCTTCGCGGCCGCGGATCCTCATTGTAGTCACTTAAGCTT |
| Mut-WIPF1-R33A/P1 | GGgcaAATGCTCTCCTTTCTGATATCAGCA |
| Mut-WIPF1-R33A/P2 | AAGGAGAGCATTtgcCCCAGCCTGCTCTGTCTTATTC |
| Mut-WIPF1-R54A/P1 | CAATGACgcaAGTGCACCAATACTGGACAAACC |
| Mut-WIPF1-R54A/P2 | GTGCACTtgcGTCATTGGTGACCGTCTTCTTTAG |

# Supplementary Table S2

Antibodies for western blot (WB), Immunoprecipitation (IP), immunofluorescence (IF) and immunohistochemistry staining (IHC).

| Primary antibody | Vendor | Origin | Dilution |
| --- | --- | --- | --- |
| WIPF1 | Thermo Fisher Scientific | Carlsbad, CA, USA | WB 1:1000; IF 1:200; IHC 1:50 |
| WIPF1 | Santa Cruz | CA, USA | IP 2μg per 500μg of total protein (1ml of cell lysate) |
| β-actin | Beyotime | China | WB 1:2000 |
| CK7 | Proteintech | Chicago, IL, USA | IF 1:1000 |
| HLA-G | Proteintech | Chicago, IL, USA | IF 1:400; WB: 1/2000 |
| VIM | Proteintech | Chicago, IL, USA | WB 1:1000; IF 1:200 |
| ITGA5 | Abcam | Cambridge, UK | WB: 1:1000 |
| MMP2 | Proteintech | Chicago, IL, USA | IF 1:200; WB: 1/1000 |
| ACTN4 | Proteintech | Chicago, IL, USA | WB 1:1000; IF 1:200; IP 2μg per 500μg of total protein (1ml of cell lysate) |
| IgG-Mo | Beyotime | China | IP 2μg per 500μg of total protein (1ml of cell lysate); IF 1:200 |
| IgG-Ra | Beyotime | China | IP 2μg per 500μg of total protein (1ml of cell lysate); IF 1:200 |
| Cortactin | Santa Cruz | CA, USA | IF 1:50 |
| VCL | Wanleibio | China | IF 1:50 |
| PL1 | Santa Cruz | CA, USA | IHC 1:50 |
| TLN1 | Santa Cruz | CA, USA | IF 1:50 |
| ITGA6 | Thermo Fisher | Carlsbad, CA, USA | MACS: 0.3 μg in a total volume of 110 μl |
| YAP | Santa Cruz | CA, USA | IF: 1:50: WB: 1:500 |
| TEAD4 | Abcam | Cambridge, UK | IF: 1:200: WB: 1:1000 |

| Secondary antibody | Vendor | Origin | Dilution ratio |
| --- | --- | --- | --- |
| Anti-mouse Alexa 488 | Thermo Fisher Scientific | Carlsbad, CA, USA | IF: 1:1000 |
| Anti-rabbit Alexa 568 | Thermo Fisher Scientific | Carlsbad, CA, USA | IF: 1:1000 |
| Anti-rabbit Alexa 647 | Thermo Fisher Scientific | Carlsbad, CA, USA | IF: 1:1000 |
| Anti-mouse Alexa 647 | Yeasen | China | IF: 1:200 |
| Anti-Rabbit IgG HRP | Thermo Fisher Scientific | Carlsbad, CA, USA | WB: 1:10000 |
| Anti-mouse IgG HRP | Thermo Fisher Scientific | Carlsbad, CA, USA | WB: 1:10000 |
| Anti-Rabbit IgG HRP (Heavy Chain Avoidance) | Abmart | China | WB: 1:2000 |

# Supplementary table S3

WIPF1-interact proteins

| Protein | Annotation | PC | UP | CP |
| --- | --- | --- | --- | --- |
| ACTB | Actin, cytoplasmic 1 | 22 | 15 | 45.9% |
| KRT6A | Keratin, type II cytoskeletal 6A | 11 | 11 | 17.0% |
| ACTC1 | Actin, alpha cardiac muscle 1 | 16 | 10 | 25.7% |
| ACTN4 | Alpha-actinin-4 | 5 | 5 | 5.5% |
| DBN1 | Drebrin 1 | 5 | 5 | 6.6% |
| ACTN1 | Alpha-actinin-1 | 4 | 4 | 4.4% |
| HBA1 | Hemoglobin subunit alpha | 4 | 4 | 29.0% |
| H2BC9 | Histone cluster 1 h2b family member h | 3 | 3 | 16.7% |
| MYH14 | Myosin heavy chain 14 | 3 | 3 | 1.6% |
| KRT13 | Keratin, type I cytoskeletal 13 | 3 | 3 | 18.2% |
| HIST1H2AB | Histone cluster 1 h2a family member b | 3 | 3 | 17.7% |
| IGH | Immunoglobulin heavy variable 4-38-2-like | 3 | 2 | 3.2% |
| ERICH3 | Glutamate-rich protein 3 | 2 | 2 | 0.9% |
| VIM | Vimentin | 2 | 2 | 4.7% |
| S100A8 | Protein S100-A8 | 2 | 2 | 19.4% |
| WIPF1 | WAS/WASL-interacting protein family member 1 | 2 | 2 | 4.2% |
| GJA10 | Gap junction alpha-10 protein | 1 | 1 | 1.2% |
| ITIH3 | Inter-alpha-trypsin inhibitor heavy chain H3 | 1 | 1 | 1.1% |
| CXCR4 | C-X-C chemokine receptor type 4 | 1 | 1 | 1.4% |
| ND4L | Mitochondrially encoded nadh:ubiquinone oxidoreductase core subunit 4l | 1 | 1 | 22.5% |
| RRBP1 | Ribosome-binding protein 1 | 1 | 1 | 1.7% |
| WASL | Wasp like actin nucleation promoting factor | 1 | 1 | 1.6% |
| FAM3C | FAM3 metabolism regulating signaling molecule C | 1 | 1 | 3.1% |
| PSD4 | PH and SEC7 domain-containing protein 4 | 1 | 1 | 0.6% |
| GAP43 | Growth associated protein 43 | 1 | 1 | 2.5% |
| MROH2A | Maestro heat-like repeat-containing protein family member 2a | 1 | 1 | 0.8% |
| TLN1 | Talin-1 | 1 | 1 | 0.6% |
| RIMS1 | Regulating synaptic membrane exocytosis protein 1 | 1 | 1 | 0.5% |
| CTBP2 | C-terminal-binding protein 2 | 1 | 1 | 1.4% |
| HIST1H4J | Histone cluster 1 H4 family member j | 2 | 1 | 11.7% |
| EFHD1 | EF-hand domain-containing protein D1 | 1 | 1 | 4.2% |
| CDY2A | Cadherin related 23 | 1 | 1 | 1.1% |
| CDH23 | Glypican-6 | 1 | 1 | 27.6% |
| GPC6 | DNA mismatch repair protein Msh6 | 1 | 1 | 1.3% |
| GTBP | Glucose-6-phosphate isomerase | 1 | 1 | 0.7% |
| GPI | Thymosin beta 4, x-linked | 1 | 1 | 4.3% |
| TMSB4X | Dedicator of cytokinesis protein 4 | 1 | 1 | 20.6% |
| DOCK4 | EH domain-containing protein 3 | 1 | 1 | 0.8% |
| EHD3 | Thyroid hormone receptor-associated protein 3 | 1 | 1 | 1.1% |
| THRAP3 | Dr1-associated corepressor | 1 | 1 | 1.0% |
| DRAP1 | FERM domain-containing protein 4A | 1 | 1 | 4.5% |
| FRMD4A | Afadin | 1 | 1 | 1.5% |
| V5-4 | NADH-ubiquinone oxidoreductase chain 5 | 1 | 1 | 10.1% |
| AFDN | Pyroglutamylated RFamide peptide receptor | 1 | 1 | 0.3% |
| NADH5 | Plectin | 1 | 1 | 1.0% |
| QRFPR | Lipopolysaccharide-responsive and beige-like anchor protein | 1 | 1 | 1.9% |
| PLEC | Lyn proto-oncogene, src family tyrosine kinase | 1 | 1 | 1.0% |
| LRBA | Vacuolar protein sorting-associated protein 13d | 1 | 1 | 0.3% |
| LYN | Kinetochore protein Spc25 | 1 | 1 | 1.2% |
| VPS13D | Kalirin | 1 | 1 | 2.6% |
| SPC25 | Disks large homolog 5 | 1 | 1 | 3.6% |
| RAMACL | Thousand and one amino acid protein kinase | 1 | 1 | 5.1% |
| KALRN | Zinc finger cchc-type containing 11 | 1 | 1 | 0.3% |
| DLG5 | Cholesterol 7-alpha-monooxygenase | 1 | 1 | 0.4% |
| TAOK2 | Ubiquitin carboxyl-terminal hydrolase isozyme L5 | 1 | 1 | 0.4% |
| TUT4 | Immunoglobulin lambda-like polypeptide 1 | 1 | 1 | 0.7% |
| CYP7A1 | Myoglobin | 1 | 1 | 1.8% |
| UCHL5 | ATP-dependent DNA helicase Q5 | 1 | 1 | 10.1% |
| IGLL1 | Pyruvate dehydrogenase protein X component, mitochondrial | 1 | 1 | 3.9% |
| MB | UPF0598 protein C8orf82 | 1 | 1 | 7.8% |
| RECQL5 | V-type proton ATPase subunit C 1 | 1 | 1 | 0.7% |
| PDHX | Cell adhesion molecule 1 | 1 | 1 | 2.0% |

Note: WIPF1-interacting protein of WIPF1-overexpression cells was found via the Co-IP and LCMSMS (nanoLC-QE) analysis. Using the Mass spectrometry matching software MASCOT, the LCMSMS data analysis provided the qualitative identification information of the WIPF1-interacting protein peptide molecules.

Abbreviations: PC: PepCount; UP: Unique PepCount; CP: Cover Percent.

# Supplementary table S4

The genes reported to participate in regulating EVTs functions list.

| Gene | AFC | AQ | Gene | AFC | AQ | Gene | AFC | AQ |
| --- | --- | --- | --- | --- | --- | --- | --- | --- |
| TMEM219 | 0.37 | 4.25E-16 | CALU | 0.63 | 2.725E-10 | TSPO | 0.92 | 4.9E-155 |
| AIF1L | 0.40 | 2.2625E-40 | PFKP | 0.63 | 2.50E-110 | FABP7 | 0.93 | 1.38E-76 |
| RPL13 | 0.40 | 8.55E-31 | CITED2 | 0.63 | 0.2 | ANGPTL4 | 0.94 | 5.54E-40 |
| S100A11 | 0.41 | 3.60E-45 | CA2 | 0.63 | 2.625E-28 | GPX4 | 0.95 | 2.20E-53 |
| YWHAZ | 0.41 | 4.70E-15 | GRB2 | 0.63 | 5.575E-36 | CLTB | 0.95 | 7.76E-71 |
| DGKZ | 0.42 | 1.008E-32 | CCR1 | 0.63 | 1.53E-41 | VGLL3 | 0.95 | 4.2E-29 |
| ITGA5 | 0.43 | 6.525E-28 | TMEM14A | 0.64 | 4.10E-14 | CALM3 | 0.96 | 3.96E-33 |
| MYCNUT | 0.43 | 3.46E-27 | PPIB | 0.64 | 8.70E-40 | MFAP5 | 0.96 | 8.36E-38 |
| MXRA8 | 0.43 | 8.06E-23 | EFNA1 | 0.64 | 3E-12 | SNAI1 | 0.97 | 1.745E-104 |
| RPL39 | 0.43 | 3.83E-46 | ADAM8 | 0.64 | 2.582E-35 | CMTM3 | 0.97 | 4.70E-58 |
| SLC39A8 | 0.44 | 2.18E-41 | AGTRAP | 0.65 | 1.07E-33 | CALD1 | 0.98 | 3.72E-41 |
| CORO1B | 0.44 | 6.55E-57 | INSIG1 | 0.65 | 1.87E-27 | TYMP | 0.98 | 7.775E-46 |
| SOX4 | 0.44 | 3.70E-56 | SDC4 | 0.65 | 7.95E-07 | CXCR6 | 0.98 | 1.21E-43 |
| KIF21A | 0.45 | 6.38E-31 | GCSH | 0.65 | 3.01E-18 | BZW2 | 0.99 | 3.00E-113 |
| SLC16A3 | 0.45 | 5.98E-37 | TNNI2 | 0.65 | 1.19E-32 | CD9 | 0.99 | 3.98E-44 |
| CAPG | 0.45 | 4.48E-07 | GPR146 | 0.66 | 2.28E-22 | CDKN1C | 1.00 | 5.00E-55 |
| ISG15 | 0.46 | 1.89E-16 | PDLIM5 | 0.66 | 3.3E-30 | LOXL1 | 1.01 | 9.04E-37 |
| B2M | 0.47 | 4.45E-43 | KRT19 | 0.66 | 5.65E-25 | EIF4EBP1 | 1.02 | 9.72E-32 |
| RPL29 | 0.48 | 3.98E-35 | PLOD1 | 0.66 | 2.78E-27 | UPP1 | 1.02 | 1.1825E-59 |
| COL27A1 | 0.48 | 1.82E-19 | MYCN | 0.67 | 5.36E-35 | TNFAIP3 | 1.03 | 9.40E-33 |
| PROCR | 0.49 | 2.078E-36 | ERO1A | 0.67 | 1.60E-66 | RFK | 1.03 | 3.60E-45 |
| S100A4 | 0.49 | 5.35E-10 | HAPLN3 | 0.68 | 1.7325E-30 | CAV2 | 1.04 | 7.3003E-71 |
| YWHAZ | 0.49 | 3.80E-13 | TAGLN2 | 0.68 | 1.92E-13 | TAP1 | 1.04 | 9.6878E-49 |
| ADAMTS1 | 0.49 | 1.5625E-61 | IL1RAP | 0.68 | 1.57E-28 | SH3GLB1 | 1.05 | 4.32E-39 |
| WIPF1 | 0.49 | 5.18E-31 | TUBB6 | 0.68 | 2.575E-18 | RPS6 | 1.07 | 2.06E-35 |
| LRRC32 | 0.50 | 1.05E-61 | PPIA | 0.69 | 2.725E-27 | MYDGF | 1.07 | 5.46E-52 |
| COTL1 | 0.50 | 1.19E-16 | SYT8 | 0.69 | 1.3625E-84 | CAPN6 | 1.07 | 4.94E-37 |
| MAP7D3 | 0.50 | 1.55E-11 | NOTUM | 0.69 | 1.895E-73 | LY6K | 1.08 | 1.74E-52 |
| KRT17 | 0.50 | 7.23E-26 | PGF | 0.69 | 1.2825E-41 | LDHA | 1.08 | 1.76E-36 |
| TMEM50A | 0.50 | 6.35E-13 | MAN1A2 | 0.70 | 1.178E-36 | PRKCDBP | 1.09 | 6.72E-35 |
| CNPY3 | 0.51 | 4.84E-37 | ANXA4 | 0.70 | 1.69E-33 | S100A6 | 1.09 | 3.55E-105 |
| RPL18A | 0.51 | 1.73E-35 | ID3 | 0.70 | 1.40E-06 | PXN | 1.10 | 6.75E-131 |
| CD276 | 0.51 | 2.445E-41 | GAPDH | 0.70 | 2.25E-69 | TMED10 | 1.10 | 3.20E-55 |
| SYDE1 | 0.52 | 2.65E-11 | DIO2 | 0.70 | 9.03E-112 | ABRACL | 1.10 | 3.52E-63 |
| QSOX1 | 0.52 | 3.60585E-61 | ADAM12 | 0.70 | 4.725E-65 | C4orf48 | 1.11 | 9.62E-80 |
| RPS27L | 0.52 | 1.48E-26 | KDELR3 | 0.70 | 6.18E-84 | HLA-C | 1.12 | 5.55E-35 |
| LITAF | 0.53 | 7.22E-12 | VASP | 0.71 | 1.39E-31 | TOMM20 | 1.14 | 1.64327E-31 |
| SEC61G | 0.53 | 5.50E-25 | GCM1 | 0.71 | 1.28E-47 | MT1G | 1.14 | 1.222E-66 |
| RABAC1 | 0.53 | 4.30E-35 | CLDN19 | 0.71 | 1.4325E-41 | IGFBP3 | 1.14 | 3.62E-39 |
| XBP1 | 0.53 | 2.25E-19 | GLIPR1 | 0.71 | 0.00000464 | FAM110A | 1.15 | 1.27E-87 |
| LAIR2 | 0.53 | 7.55E-25 | IL2RB | 0.72 | 6.92E-39 | AFAP1 | 1.16 | 2.22E-65 |
| PLK2 | 0.53 | 8.60E-20 | SLCO4A1 | 0.72 | 2.88E-45 | MT1H | 1.17 | 1.5825E-32 |
| CSTB | 0.54 | 5.28E-29 | ERBB2 | 0.72 | 2.52E-33 | COLGALT1 | 1.19 | 1.56E-24 |
| FBLIM1 | 0.54 | 2.03E-39 | FABP5 | 0.73 | 2.8E-31 | HTRA4 | 1.19 | 3.775E-60 |
| CALR | 0.54 | 3.275E-29 | IMPA2 | 0.73 | 2.76E-21 | MT1X | 1.19 | 1.046E-12 |
| WIPI1 | 0.55 | 4.60E-70 | AP3S1 | 0.73 | 3.66E-08 | BCAR4 | 1.21 | 8.58E-22 |
| HLA-G | 0.55 | 1.95E-05 | STRA13 | 0.73 | 2.32E-37 | GLRX | 1.24 | 6.78E-38 |
| ACAN | 0.55 | 4.24E-13 | PLOD2 | 0.74 | 5.60E-19 | SRI | 1.25 | 5.475E-80 |
| RPS24 | 0.55 | 3.93E-50 | RGS16 | 0.74 | 4.42E-18 | TIMP1 | 1.26 | 2.24E-78 |
| TMSB10 | 0.55 | 1.58E-46 | C12orf75 | 0.74 | 5.21E-23 | ASCL2 | 1.27 | 1.796E-73 |
| MORF4L2 | 0.55 | 5.15E-25 | CHCHD10 | 0.75 | 6.35E-34 | SSR3 | 1.27 | 3.22E-29 |
| TMEM256 | 0.55 | 1.65E-46 | ACTB | 0.76 | 8.15E-74 | KRT8 | 1.29 | 2.34E-61 |
| RPL28 | 0.56 | 5.58E-54 | COL4A1 | 0.76 | 4.38E-201 | GM2A | 1.31 | 4.04001E-73 |
| SDF4 | 0.56 | 1.73E-22 | CAP1 | 0.76 | 1.10E-16 | GALNT2 | 1.31 | 7.84E-84 |
| KLF6 | 0.56 | 6.05E-48 | TFPI | 0.76 | 7.40E-21 | PLAC8 | 1.33 | 1.015E-47 |
| PPP1R14B | 0.56 | 1.42E-14 | OSR2 | 0.77 | 9.00E-92 | JUNB | 1.34 | 6.28E-59 |
| WFS1 | 0.56 | 3.025E-29 | CD63 | 0.78 | 3.38E-40 | TUBA1C | 1.34 | 7.18E-49 |
| ADA | 0.57 | 4.88E-26 | LY6E | 0.79 | 1.13E-50 | MGST1 | 1.38 | 2.58E-36 |
| PDIA6 | 0.57 | 6.18001E-26 | CNN2 | 0.79 | 1.492E-35 | EBI3 | 1.39 | 5.76E-87 |
| HN1 | 0.57 | 1.48E-07 | SPON2 | 0.79 | 2.32E-25 | ISG20 | 1.43 | 1.37E-56 |
| KRTCAP2 | 0.57 | 4.56E-27 | PON2 | 0.80 | 1.73E-81 | HPCAL1 | 1.44 | 4.42E-32 |
| N4BP3 | 0.57 | 2.575E-12 | RPL12 | 0.80 | 1.8425E-66 | PLIN2 | 1.45 | 9.98E-50 |
| SH3BGRL3 | 0.57 | 1.06E-21 | LPCAT1 | 0.80 | 4.725E-82 | LHFP | 1.49 | 2.78E-32 |
| PYGL | 0.58 | 4.63E-45 | MYADM | 0.81 | 2.52E-27 | MTDH | 1.49 | 3.32E-70 |
| RALB | 0.58 | 4.24E-11 | KRT10 | 0.83 | 5.02E-32 | SLC25A19 | 1.49 | 8.57566E-61 |
| MFAP2 | 0.58 | 1.23075E-55 | SH3BP5 | 0.83 | 1.45E-13 | HLA-E | 1.55 | 2.22E-48 |
| RPS17 | 0.58 | 1.98E-53 | ARHGDIB | 0.83 | 4.15E-32 | ADAM15 | 1.57 | 1.73E-200 |
| TCF7L2 | 0.58 | 1.43E-50 | PTPRF | 0.83 | 1.05E-64 | ID1 | 1.58 | 1.44E-158 |
| GPX8 | 0.59 | 8.825E-33 | LIMA1 | 0.85 | 2.08E-44 | C1QTNF6 | 1.58 | 7.6E-50 |
| FLNB | 0.59 | 6.38E-10 | MT1E | 0.86 | 9.40E-32 | MT1F | 1.61 | 6.375E-66 |
| FHL2 | 0.59 | 1.82E-43 | ABCA7 | 0.86 | 0.001773168 | LAMA4 | 1.68 | 1.45E-95 |
| KCTD12 | 0.59 | 2.50E-01 | ACTG1 | 0.87 | 1.222E-52 | CSF1R | 1.68 | 1.83E-153 |
| ENG | 0.59 | 5.94E-39 | VKORC1 | 0.88 | 1.72E-134 | TGFB1 | 1.70 | 8.78E-48 |
| SELM | 0.60 | 1.632E-22 | CEBPB | 0.88 | 2.55E-85 | EFHD2 | 1.71 | 4.475E-38 |
| RPL9 | 0.60 | 1.025E-26 | BRI3 | 0.88 | 3.60E-37 | COL4A2 | 1.74 | 3.40E-155 |
| TPM1 | 0.60 | 1.59E-23 | HPGD | 0.88 | 2.55E-172 | TSC22D3 | 1.76 | 9.64E-57 |
| RPL37A | 0.61 | 1.13E-38 | MIF | 0.89 | 7.5E-68 | HSPA2 | 1.78 | 1.27E-14 |
| PRSS8 | 0.61 | 2.52E-21 | NRIP1 | 0.90 | 5.38E-17 | HEG1 | 1.78 | 9E-91 |
| RALBP1 | 0.61 | 3.62E-50 | EPSTI1 | 0.90 | 1.34E-22 | AXIN2 | 1.84 | 7.475E-160 |
| CAV1 | 0.62 | 1.92E-58 | LGALS1 | 0.90 | 5.85E-64 | IGSF8 | 1.87 | 5.575E-40 |
| AES | 0.62 | 7.06E-20 | SERPINB6 | 0.91 | 5.00E-55 | RAB8B | 1.91 | 2.30E-68 |
| HLA-B | 0.62 | 1.49E-109 | IFITM3 | 0.91 | 2.1725E-09 | NPC2 | 2.11 | 2.70E-275 |
| LAPTM4A | 0.62 | 8.45E-59 | APOC1 | 0.91 | 3.23E-117 | FXYD5 | 2.16 | 5.7E-163 |
| RPL37 | 0.63 | 8.15E-50 | FOSL2 | 0.91 | 5.60E-110 | HMOX1 | 2.30 | 4.15E-81 |
| FSTL3 | 0.63 | 1.02E-33 | CKS1B | 0.92 | 5.98E-16 |  |  |  |

Abbreviations: AFC: Average of log2FC in EVTs subtypes; AQ: Average of adjusted *P* value in EVTs subtypes.
