# Supplementary material for: Unraveling the role of the WIPF1/ACTN4 complex in podosome formation of human placental EVTs: Insights into recurrent spontaneous abortion
Source: Genes Dis. 2025 May 2;12(6):101665. doi: 10.1016/j.gendis.2025.101665 (PMC12357052; doi:10.1016/j.gendis.2025.101665)
Supplement: Multimedia component 2 [file mmc2.docx]

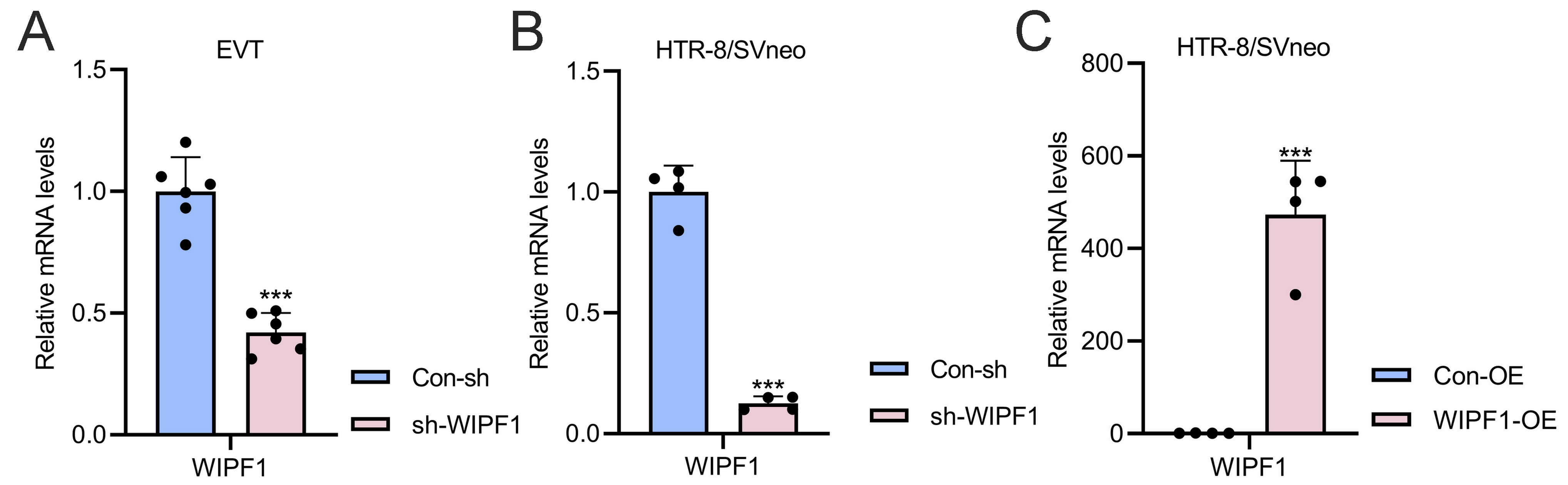
 **Figure S1** mRNA levels of WIPF1 in lentivirus-infected cells. **(A)** mRNA levels of WIPF1 in sh-WIPF1 extravillous trophoblasts (EVTs), relative to the mRNA levels of GADPH. ^***^*P* < 0.001. **(B)** mRNA levels of WIPF1 in sh-WIPF1 HTR-8/SVneo cells, relative to the mRNA levels of GADPH. ^***^*P* < 0.001. **(C)** mRNA levels of WIPF1 in Con-OE and WIPF1-OE HTR-8/SVneo cells, relative to the mRNA levels of GADPH. ^***^*P* < 0.001. WIPF1, WAS/WASL interacting protein family member 1.


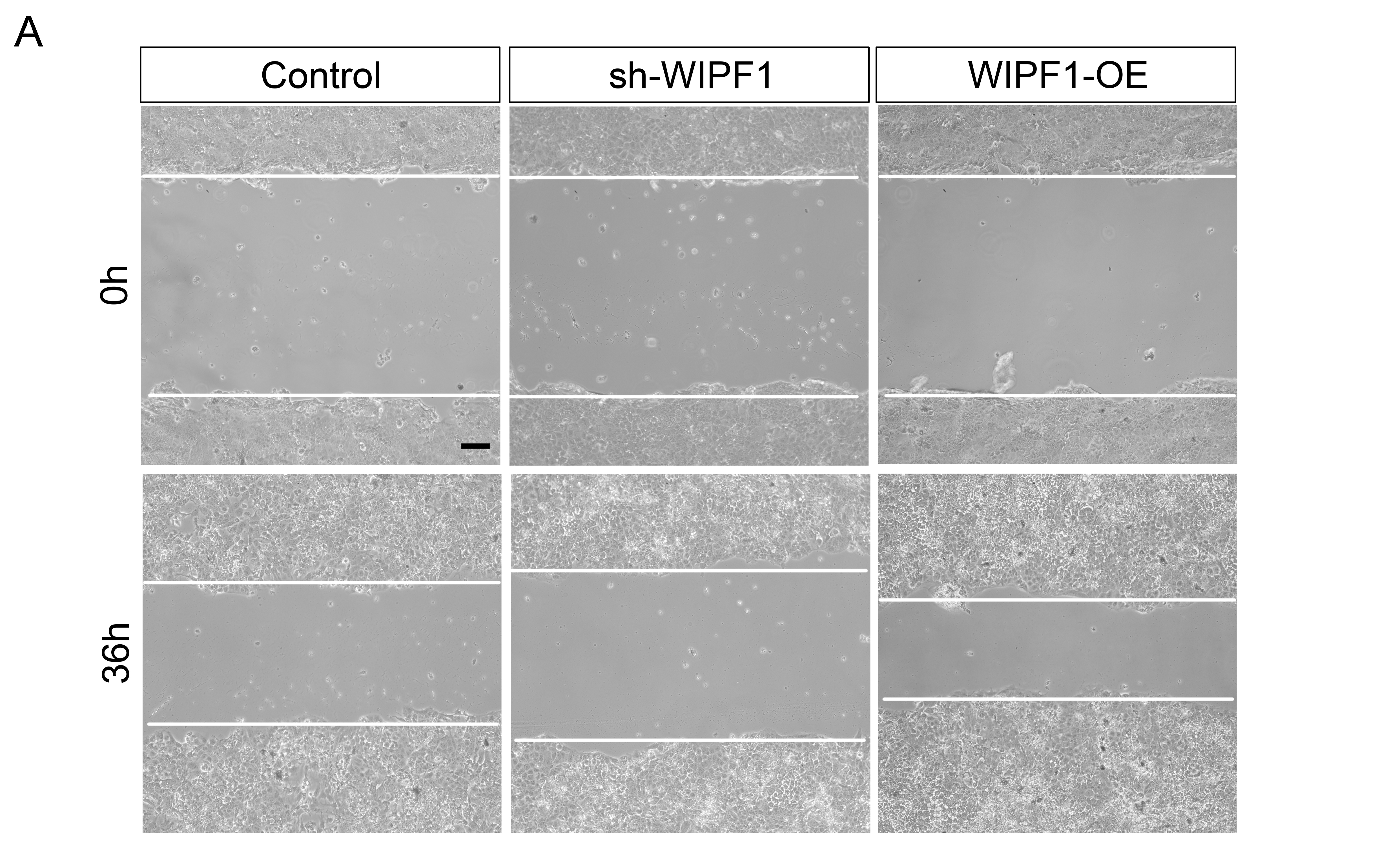


**Figure S2** WIPF1 promotes the migration of JEG-3 cells. The images show the migration abilities of the wild-type JEG-3 cells, sh-WIPF1 JEG-3 cells, and WIPF1-OE JEG-3 cells after 0 h and 36 h of treatment. Scale bars, 100 μm. WIPF1, WAS/WASL interacting protein family member 1.


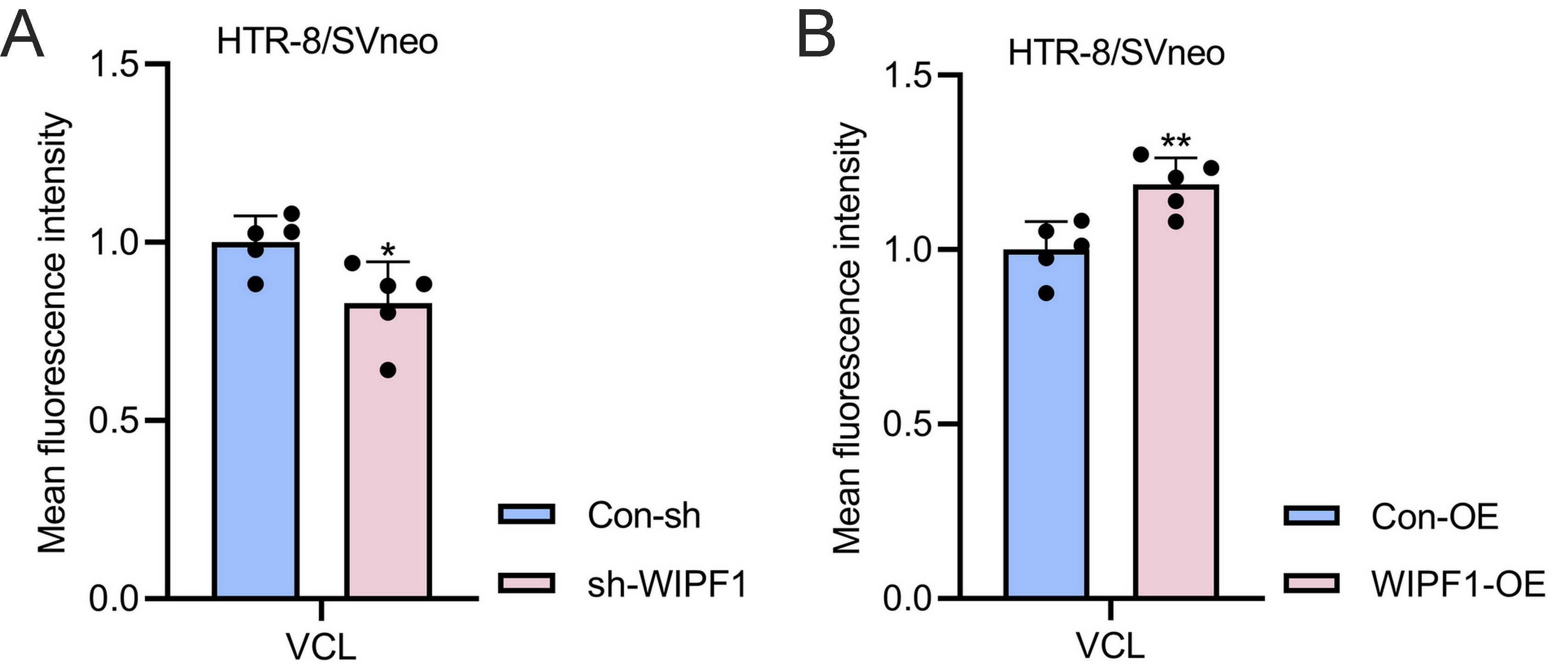


**Figure S3** Expression of vinculin (VCL) in WIPF1-sh and WIPF1-OE HTR-8/SVneo cells. **(A, B)** Statistical plot of mean fluorescence intensity of VCL in Con-sh, WIPF1-sh, Con-OE, and WIPF1-OE HTR-8/SVneo cells. ^*^*P* < 0.05 and ^**^*P* < 0.01. WIPF1, WAS/WASL interacting protein family member 1.


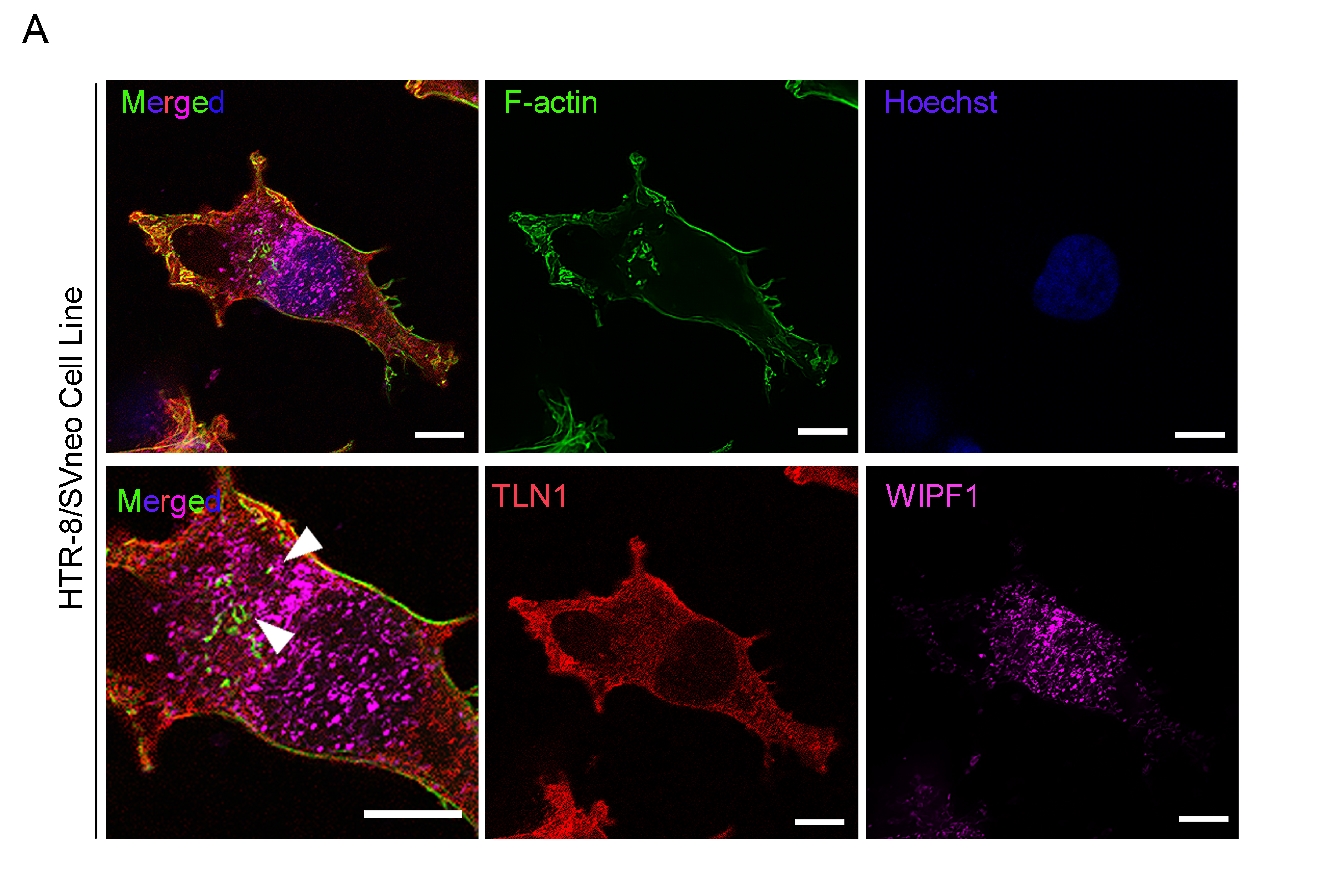


**Figure S4** Visualization of WIPF1, TLN1, and F-actin in HTR-8/SVneo Cells. The immunofluorescence images show the colocalization of WIPF1, TLN1, and phalloidin in HTR-8/SVneo cells. Scale bars, 10 μm. WIPF1, WAS/WASL interacting protein family member 1; TLN1, talin 1.


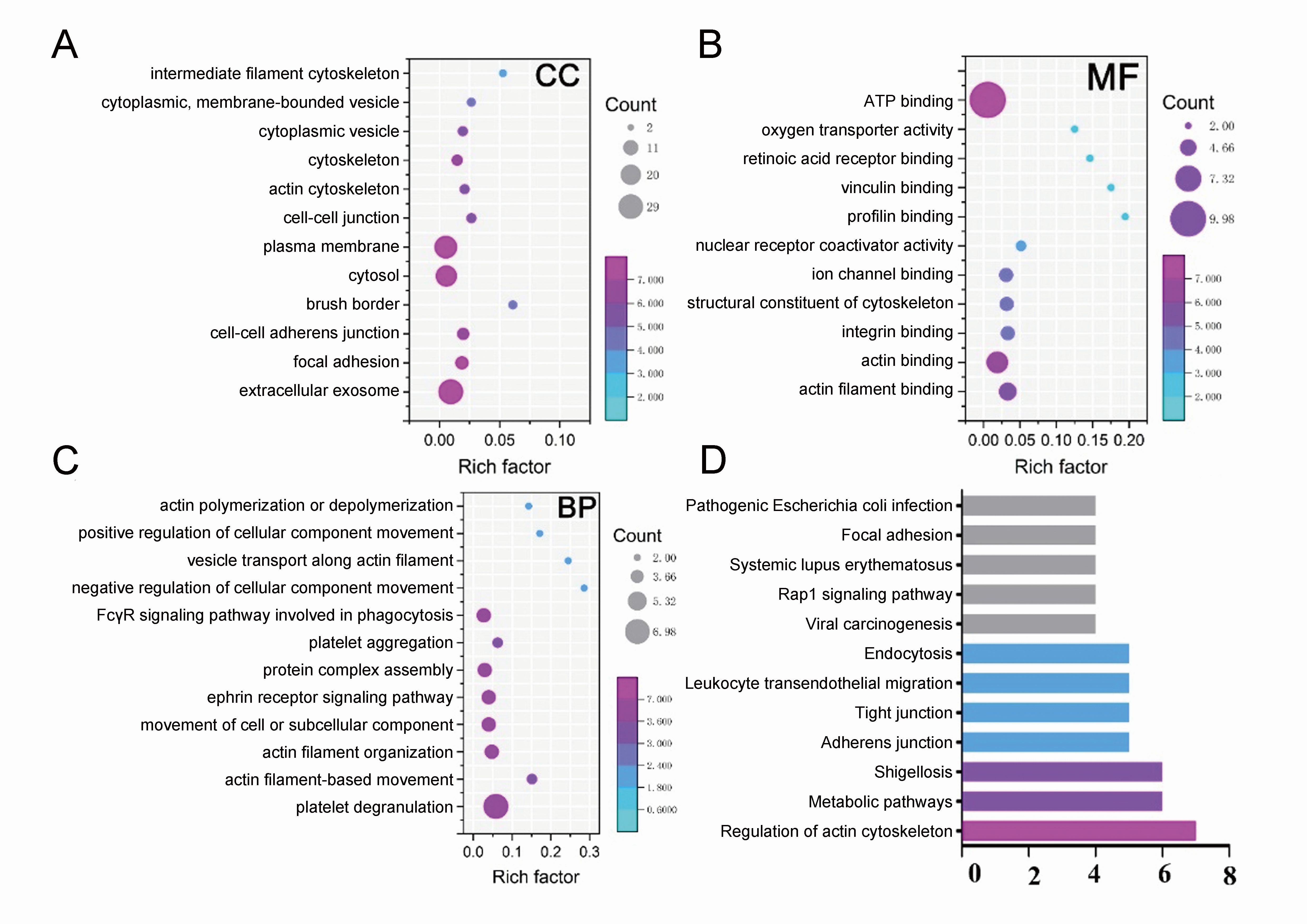


**Figure S5** Co-immunoprecipitation-mass proteomic analysis of WIPF1 interactors in HTR-8/SVneo cell line. **(A–C)** Gene Ontology (GO) analysis of WIPF1 specific interaction proteins. The bubble chart represents the rich factor and count of WIPF1 interactome via mass spectrometry and in WIPF1 immunoprecipitates. BP, biological process. MF, molecular function. CC, cellular component. The right gradient means −log_10_ (*P*-value). **(D)** The bar chart for the Kyoto Encyclopedia of Genes and Genomes (KEGG) analysis of WIPF1 interactome. WIPF1, WAS/WASL interacting protein family member 1.


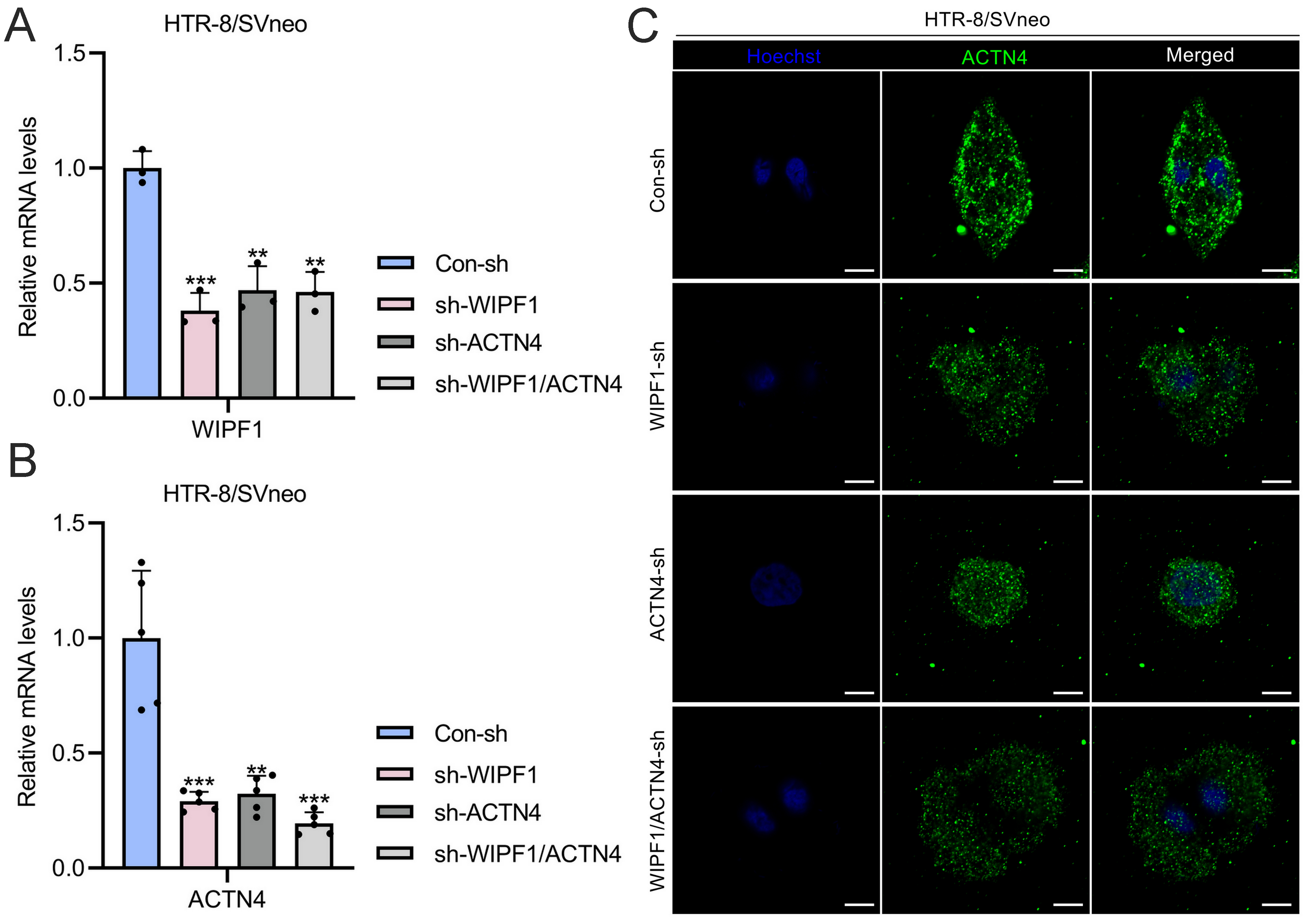


**Figure S6** Expression of ACTN4 after single or double knockdown of WIPF1 and ACTN4 in HTR-8/SVneo cells. **(A)** mRNA levels of ACTN4 after single and double knockdown of WIPF1 and ACTN4 in HTR-8/SVneo cells, relative to the mRNA levels of GADPH. ^**^*P* < 0.01 and ^***^*P* < 0.001. **(B)** mRNA levels of WIPF1 after single and double knockdown of WIPF1 and ACTN4 in HTR-8/SVneo cells, relative to the mRNA levels of GADPH. ^**^*P* < 0.01. **(C)** Immunofluorescence images of the expression of ACTN4 (green) after single or double knockdown of WIPF1 and ACTN4 in HTR-8/SVneo cells. The blue colors represent nuclei stained with Hoechst. Scale bars, 10 μm. ACTN4, alpha-actinin 4; WIPF1, WAS/WASL interacting protein family member 1.


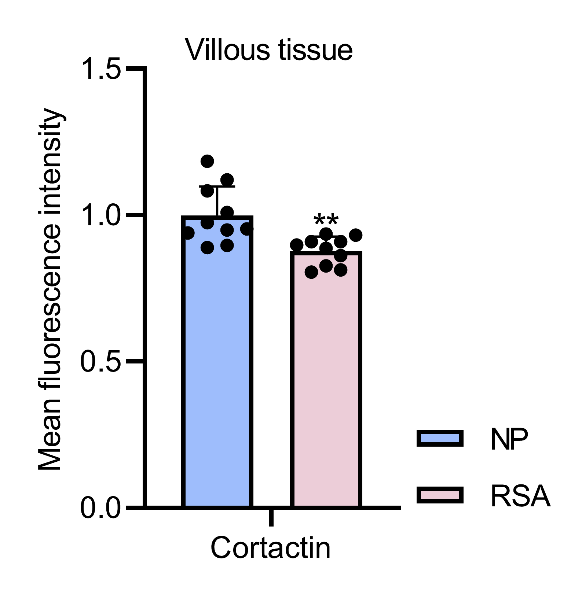


**Figure S7** Expression of cortactin in recurrent spontaneous abortion (RSA) and normal pregnancy (NP) early pregnancy villous tissues. Statistical plot of mean fluorescence intensity (MFI) of cortactin in RSA and NP early pregnancy villous tissues, corresponding to Figure 7F. au is the unit of the average fluorescence intensity. ^**^*P* < 0.01.


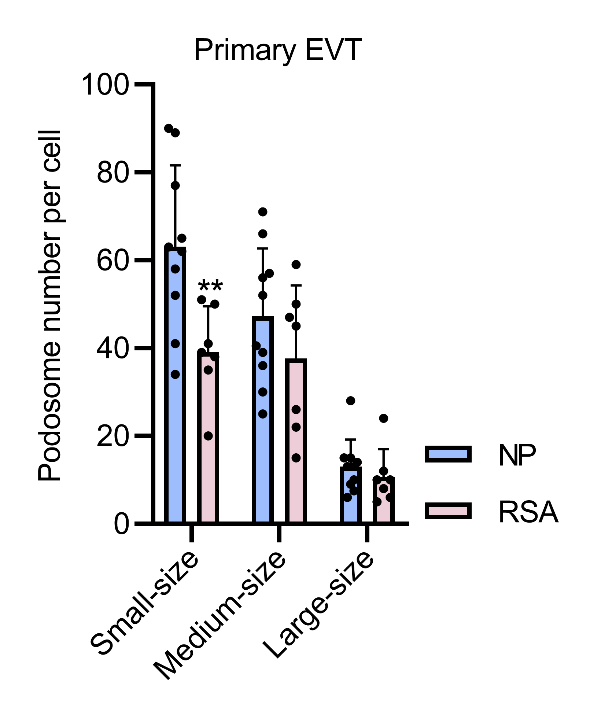


**Figure S8** Distribution of small-, medium-, and large-sized podosomes in normal pregnancy (NP) and recurrent spontaneous abortion (RSA) extravillous trophoblasts (EVTs). The number of podosomes in NP and RSA EVTs (^**^*P* < 0.01). Podosome size distribution was categorized into small (0.2–0.5 μm²), medium (0.5–1.5 μm²), and large (1.5–3 μm²) groups. The distribution of these podosomes in the primary EVTs from both RSA and NP groups was compared. A significant difference in the number of podosomes across different sizes was observed between the RSA and NP groups, with variations in the size distribution patterns between the two groups.


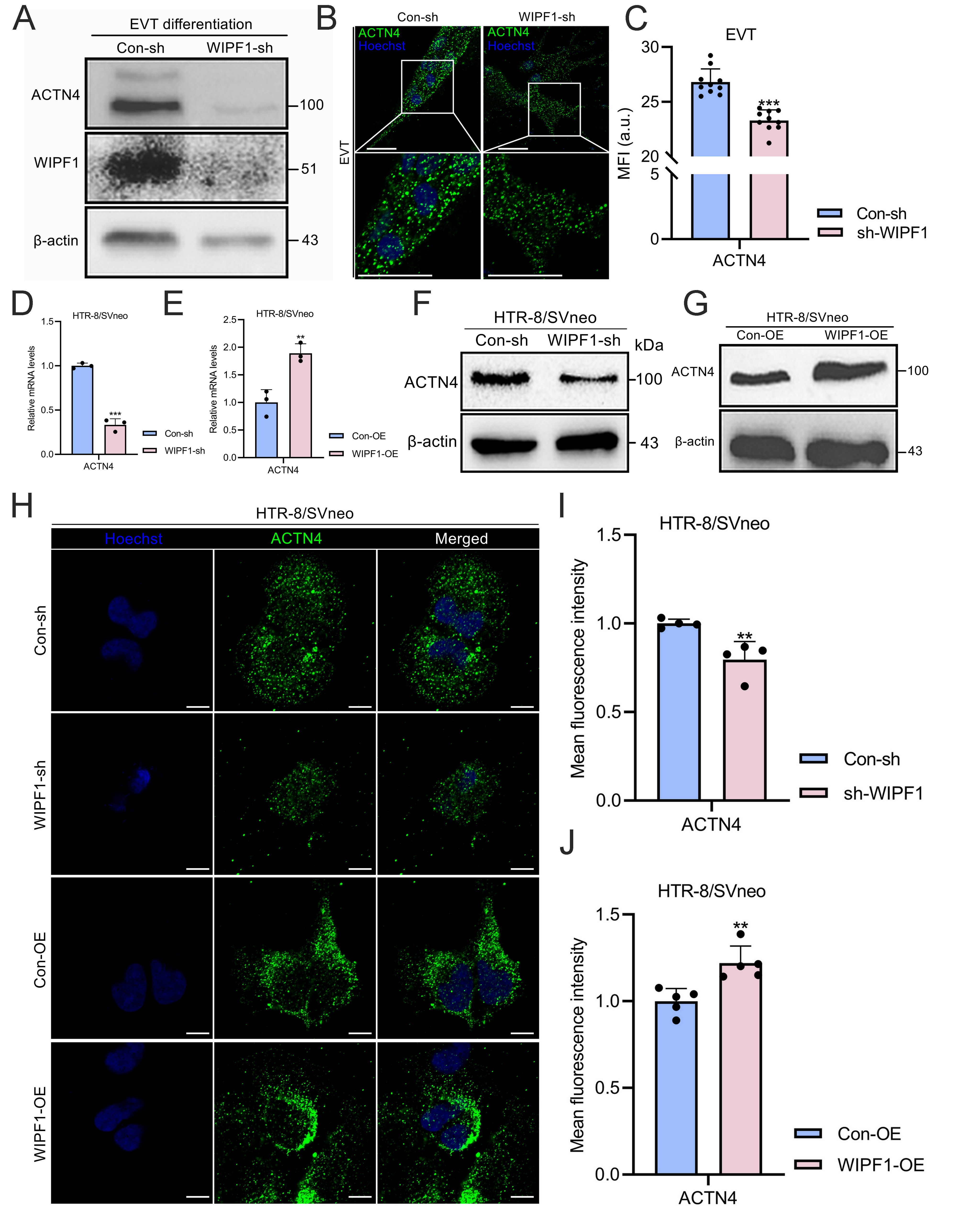


**Figure S9** Expression of ACTN4 in WIPF1-modified HTR-8/SVneo cells and human trophoblast stem cell (hTSC)-derived extravillous trophoblasts (EVTs). **(A)** Protein levels of ACTN4 and WIPF1 in the Con-sh and WIPF1-sh cells in EVT differentiation. **(B)** Immunofluorescence images of the expression of ACTN4 (green) in sh-WIPF1 EVT differentiation. The blue colors represent nuclei stained with Hoechst. Scale bars, 25 μm. **(C)** Statistical plot of mean fluorescence intensity (MFI) of ACTN4 in sh-WIPF1 EVT differentiation. ^***^*P* < 0.001. **(D, E)** mRNA levels of matrix metallopeptidase 2 (MMP2) in sh-WIPF1 and WIPF1-OE HTR-8/SVneo cells, relative to the mRNA levels of GADPH. ^**^*P* < 0.01. **(F, G)** Protein levels of ACTN4 in WIPF1-sh and WIPF1-OE HTR-8/SVneo cells. **(H)** Immunofluorescence images of the expression of ACTN4 (green) in Con-sh, WIPF1-sh, Con-OE, and WIPF1-OE HTR-8/SVneo cells. The blue colors represent nuclei stained with Hoechst. Scale bars, 10 μm. **(I, J)** Statistical plot of the mean fluorescence intensity of ACTN4 in Con-sh, WIPF1-sh, Con-OE, and WIPF1-OE HTR-8/SVneo cells, normalized to the mean value of the respective control group. ^**^*P* < 0.01. ACTN4, alpha-actinin 4; WIPF1, WAS/WASL interacting protein family member 1.
